# Efficient results on fractional Langevin-Sturm-Liouville problem via generalized Caputo-Atangana-Baleanu derivatives

**DOI:** 10.1371/journal.pone.0311141

**Published:** 2024-10-02

**Authors:** Sabri T. M. Thabet, Abdelatif Boutiara, Mohammad Esmael Samei, Imed Kedim, Miguel Vivas-Cortez

**Affiliations:** 1 Department of Mathematics, Saveetha School of Engineering, Saveetha Institute of Medical and Technical Sciences, Saveetha University, Chennai, Tamil Nadu, India; 2 Department of Mathematics, Radfan University College, University of Lahej, Lahej, Yemen; 3 Department of Mathematics, College of Science, Korea University, Seoul, Republic of Korea; 4 Department of Mathematics and Computer Sciences, University of Ghardaia, Ghardaia, Algeria; 5 Department of Mathematics, Faculty of Science, Bu-Ali Sina University, Hamedan, Iran; 6 Department of Mathematics, College of Science and Humanities in Al-Kharj, Prince Sattam Bin Abdulaziz University, Al-Kharj, Saudi Arabia; 7 Faculty of Exact and Natural Sciences, School of Physical Sciences and Mathematics, Pontifical Catholic University of Ecuador, Sede Quito, Ecuador; Kwame Nkrumah University of Science and Technology, GHANA

## Abstract

In this paper, we investigate the generalized Langevin-Sturm-Liouville differential problems involving Caputo-Atangana-Baleanu fractional derivatives of higher orders with respect to another positive, increasing function denoted by *ρ*. The fixed point theorems in the framework of Kransnoselskii and Banach are utilized to discuss the existence and uniqueness of the results. In addition, the stability criteria of Ulam-Hyers, generalize Ulam-Hyers, Ulam-Hyers-Rassias, and generalize Ulam-Hyers-Rassias are investigated by non-linear analysis besides fractional calculus. Finally, illustrative examples are reinforced by tables and graphics to describe the main achievements.

## 1 Introduction

The powerful effect of fractional derivatives and integrals in checking the existence of solutions for differential equations (DEs) is very impressive, see books [1–4] and references therein. In addition, the combination of nonlinear analysis and fixed point theorems (FPTs) with nonlinear fractional differential equations (FDEs) along with their stability analysis has been well presented by the authors in the published articles [5–8].

In 2016, Atangana and Baleanu introduced the following new fractional derivative with non-local and non-singular kernel

DABCaϑω(υ)=Λ(ϑ)1-ϑ∫aυω′(s)Eϑ(-ϑ(υ-s)ϑ1-ϑ)ds, (1)

presented some useful properties of the derivative and applied to solve the fractional heat transfer model [9]. In 2018, Baleanu and Fernandez [10] introduced ABC fractional derivatives with respect to another function *ρ* for order belongs to the interval (0, 1). Recently in 2023, the authors [11] extended it to higher fractional order with respect to another function. Thabet *et al.* studied a new category of implicit *ρ*-Caputo-Atangana-Baleanu (ρ-ABC) fractional pantograph systems as form

DABCaϑ1,ρω(υ)=g(υ,ω(υ),ω(δυ),DABCaϑ,ρω(υ)),υ∈[a,b],0<δ<1, (2)

under the conditions $\omega \left(a\right)=\eta_{1}$, $\omega \left(b\right)=\eta_{2}\int_{a}^{b}ϒ\left(\xi ,\omega \left(\xi \right)\right)\;d\xi$, with $\eta_{i}\in R$, 1 < *ϑ* ≤ 2 and continuous functions g [12]. In 2024, Rezapour et al. [13] used the topology degree approach to study implicit FDE with three-point boundary conditions via the same ρ-ABC derivatives.

The Langevin-Sturm-Liouville problems have attracted attention of many researchers, and used to describe dynamical systems in engineering and physics fields. Additionally, these equations are studied under various fractional derivatives by several approaches. For example, see these articles [14–17].

Inspired by the above research works, the current article focuses on studying the qualitative properties of the solutions for the ρ-ABC fractional generalized Langevin-Sturm-Liouville problem (FGLSLP) of the form:

{DABCaϑ1,ρ[μ(υ)DABCaϑ2,ρ+γ(υ)]ω(υ)=g(υ,ω(υ)),υ∈J=[a,b],ω(a)=η1,[μ(a)DABCaϑ2,ρ+γ(a)]ρ(k)ω(a)=ξk,0≤k≤m, (3)

where $\eta_{1},\xi_{k}\in R\;\left(k=0,1,\ldots ,m\right),m\in N$, DABCaϑ1,ρ and DABCaϑ2,ρ denote the ρ-ABC fractional derivatives of arbitrary orders *ϑ*_1_ ∈ (*m*, *m* + 1] and *ϑ*_2_ ∈ (0, 1] respectively, the functions γ∈C(J,R), g∈C(J×R,R), μ∈C(J,R\{0}), with |*μ*(*υ*)| ≥ λ > 0 and $\rho :J\to R^{+}$ be a non-decreasing and non-negative function with $\rho \left(u\right)\in C=C^{m}\left(\left(a,b\right),R^{+}\right)$, such that *ρ*′(*υ*) ≠ 0 on J.

The innovation and contributions of this work are that the FGLSLP (3) is new and for the first time studied under the ρ-ABC fractional derivatives of higher order. Moreover, the FGLSLP (3) can be reduced to the ABC fractional derivatives sense if *ρ*(*υ*) = *υ*. Furthermore, the FGLSLP (3) can be reduced to the Langevin problem if *μ*(*υ*) = 1 and γ(υ)=δ∈R, as follows:

{DABCaϑ1,ρ[DABCaϑ2,ρ+δ]ω(υ)=g(υ,ω(υ)),υ∈J=[a,b],ω(a)=η1,[DABCaϑ2,ρ+δ]ρ(k)ω(a)=ξk,0≤k≤m, (4)

also returns to the Sturm-Liouville problem if *γ*(*υ*) = 0 as follows:

{DABCaϑ1,ρ[μ(υ)DABCaϑ2,ρ]ω(υ)=g(υ,ω(υ)),υ∈J=[a,b],ω(a)=η1,[μ(a)DABCaϑ2,ρ]ρ(k)ω(a)=ξk,0≤k≤m. (5)

The structure of this article is as: In Sec 2, we present the basics and background materials. In Sec 3, we prove the qualitative results by helping the classical FPTs. In Sec 4, we discuss the Ulam-Hyers (UH) stability and the Ulam-Hyers-Rassias (UHR) stability. Finally, in Sec 5, we provide numerical examples with graphics and tables to test major results.

## 2 Preliminaries

We are in a position to present several major definitions and notations. Assume that Banach space denoted by *χ* ≔ *C*(J) endowed with norm ‖*ω*‖ = sup_*υ*∈J_ |*ω*(*υ*)|.

**Definition 2.1 ([18])**. *For ϑ* > 0, *the ρ-Riemann–Liouville fractional integral of integrable function* ω:J→R *is defined as*

IRLaϑ,ρω(υ)=∫aυ(ρ^s(υ))ϑ-1ρ′(s)Γ(ϑ)ω(s)ds, (6)

*where* ${\hat{\rho }}_{s}\left(\upsilon \right)=\rho \left(\upsilon \right)-\rho \left(s\right)$ *and* Γ *is Gamma function*.

**Lemma 2.2 ([18])**. *Let* ω:J→R *and ϑ*, *μ* > 0. *Then*

1.
IRLaϑ,ρ(ρ^a(υ))μ-1=Γ(μ)Γ(ϑ+μ)(ρ^a(υ))ϑ+μ-1 ;
2.
IRLaϑ,ρIRLaμ,ρω(υ)=IRLaϑ+μ,ρω(υ) ;
3.
((1ρ(υ)ddυ)mIRLam,ρω)(υ)=ω(υ),m∈N .

**Definition 2.3 ([11])**. *Let ϑ* ∈ (*m*, *m* + 1], *ν* = *ϑ* − *m*, *m* = 0, 1, 2, …. *Then, the* ρ-ABC *fractional derivative and the* ρ-AB *fractional integral of a function* $\omega \in L^{m+1}\left(\left(a,b\right)\right)$ *are defined as*

(DABCaϑ,ρω)(υ)=(DABCaν,ρωρ(m))(υ)=Λ(ϑ-m)m+1-ϑ∫aυρ′(s)Eϑ-m(-(ϑ-m)m+1-ϑ(ρ^s(υ))ϑ-m)ωρ(m+1)(s)ds, (7)

*and*

(IABaϑ,ρω)(υ)=(IRLam,ρIABaν,ρω)(υ)=(IABaν,ρIRLam,ρω)(υ)=m+1-ϑΛ(ϑ-m)IRLam,ρω(υ)+(ϑ-m)Λ(ϑ-m)IRLaϑ,ρω(υ), (8)

*respectively, where*

$$\begin{array}{c} \omega_{\rho }^{\left(m\right)}\left(\upsilon \right)=\{\begin{array}{cc} {\left(\frac{1}{\rho^{\prime }\left(\upsilon \right)}\frac{d}{d\upsilon }\right)}^{m}\omega \left(\upsilon \right), & m\in N,\\ \omega (\upsilon ), & m=0, \end{array} \end{array}$$ (9)

$E_{\vartheta }$ *is the Mittag-Leffler function given by*

$$\begin{array}{c} E_{\vartheta }(z)=\sum_{i=0}^{\infty }\frac{z^{i}}{\Gamma (\vartheta i+1)},\;\text{Re}(\vartheta )>0,\;z\in C, \end{array}$$ (10)

Λ(*ϑ*) *satisfied* Λ(0) = Λ(1) = 1 *and is called normalization function, and* IRLam,ρ *is defined as*

(IRLam,ρω)(υ)=∫aυρ′(s)Γ(m)(ρ^s(υ))m-1ω(s)ds,υ>a. (11)

If ϑ=k∈N, then (DABCaϑ,ρω)(υ)=ωρ(k)(υ).

**Lemma 2.4 ([11]).** *Let ω* ∈ *χ, and* *ρ* ∈ *C*^*m*^(J). *For ν* = *ϑ* − *m*, *ϑ* ∈ (*m*, *m* + 1], *m* = 0, 1, 2, …, *then*

(IABaϑ,ρDABaϑ,ρω)(υ)=ω(υ)-∑k=0mek(ρ^a(υ))k,ek∈R. (12)

**Lemma 2.5 ([11])**. *Let ρ* ∈ *C*^*m*^(J), *ρ*′(*υ*) ≠ 0. *For ν* = *ϑ* − *m*, *ϑ* ∈ (*m*, *m* + 1], *α* ≥ *m* + 1, *m* = 0, 1, 2, …. *Then*,

1.
IABaϑ,ρ(ρ^a(υ))α=(m+1-ϑ)Γ(α+1)(ρ^a(υ))α+mΛ(ϑ-m)Γ(m+α+1)+(ϑ-m)Γ(α+1)(ρ^a(υ))α+ϑΛ(ϑ-m)Γ(ϑ+α+1) ;
2.
(IABaϑ,ρ1)(υ)=(m+1-ϑ)(ρ^a(υ))mΛ(ϑ-m)Γ(m+1)+(ϑ-m)(ρ^a(υ))ϑΛ(ϑ-m)Γ(ϑ+1) .

**Theorem 2.6 (Krasnoselskii’s FPT [19])**. *Let K be a Banach space, and H* ⊆ *K be a nonempty, convex, and closed set. Suppose that ψ*_1_ *and ψ*_2_ *are two mappings where* (**i**) *ψ*_1_*ζ*_1_ + *ψ*_2_*ζ*_2_ ∈ *H, for all* *ζ*_1_, *ζ*_2_ ∈ *H*; (**ii**) *ψ*_1_ *is continuous and compact*; (**iii**) *ψ*_2_ *is contraction. Then, there is* *υ* ∈ *H*, *where* *υ* = *ψ*_1_*υ* + *ψ*_2_*υ*.

## 3 Existence and uniqueness criteria

We are in a position to derive the equivalent solution of the ρ-ABC FGLSLP (3). Thus, we start to present the next lemma.

**Lemma 3.1** *Let* $q:J\to R,\vartheta_{1}\in \left(m,m+1\right],\vartheta_{2}\in \left(0,1\right]$ *and ω* ∈ *χ*. *Then, the equivalent solution form of the* ρ-ABC FGLSLP:

{DABCaϑ1,ρ[μ(υ)DABCaϑ2,ρ+γ(υ)]ω(υ)=q(υ),υ∈J,ω(a)=η1,[μ(a)DABCaϑ2,ρ+γ(a)]ρ(k)ω(a)=ξk,0≤k≤m, (13)

*is given by*

ω(υ)=η1+(1-ϑ2)Λ(ϑ2)[η1γ(a)μ(a)-ξ0μ(a)]-IABaϑ2,ρ[γ(υ)μ(υ)ω(υ)]+IABaϑ2,ρ[1μ(υ)∑k=0mξkk!(ρ^a(υ))k]+IABaϑ2,ρ[1μ(υ)IABaϑ1,ρq(υ)]. (14)

*Proof*. By taking IABaϑ1,ρ on both sides of Eq (13) and using Lemma 2.4, we get

[μ(υ)DABCaϑ2,ρ+γ(υ)]ω(υ)=∑k=0mek(ρ^a(υ))k+IABaϑ1,ρq(υ)=∑k=0mek(ρ^a(υ))k+m+1-ϑ1Λ(ϑ1-m)IRLam,ρq(υ)+(ϑ1-m)Λ(ϑ1-m)IRLaϑ1,ρq(υ). (15)

By taking *i*-th derivatives with respect to a function *ρ* where *i* = 0, 1, …, *m*, we have

[μ(υ)DABCaϑ2,ρ+γ(υ)]ρ(i)ω(υ)=∑k=imekk!(k-i)!(ρ^a(υ))k-i+m+1-ϑ1Λ(ϑ1-m)IRLam-i,ρq(υ)+(ϑ1-m)Λ(ϑ1-m)IRLaϑ1-i,ρq(υ). (16)

Now, by the condition [μ(a)DABCaϑ2,ρ+γ(a)]ρ(i)ω(a)=ξi, 0 ≤ *i* < *m*, we deduce that $e_{i}=\frac{\xi_{i}}{i!}$. Then, we obtain

[μ(υ)DABCaϑ2,ρ+γ(υ)]ω(υ)=∑k=0mξkk!(ρ^a(υ))k+IABaϑ1,ρq(υ), (17)

which implies that

DABCaϑ2,ρω(υ)=1μ(υ)∑k=0mξkk!(ρ^a(υ))k-γ(υ)μ(υ)ω(υ)+1μ(υ)IABaϑ1,ρq(υ). (18)

Next, by taking IABaϑ2,ρ on both sides of Eq (18), and via Lemma 2.4, we find

ω(υ)=IABaϑ2,ρ[1μ(υ)∑k=0mξkk!(ρ^a(υ))k-γ(υ)μ(υ)ω(υ)+1μ(υ)IABaϑ1,ρq(υ)]+c0=IABaϑ2,ρ[1μ(υ)∑k=0mξkk!(ρ^a(υ))k]-IABaϑ2,ρ[γ(υ)μ(υ)ω(υ)]+IABaϑ2,ρ[1μ(υ)IABaϑ1,ρq(υ)]+c0, (19)

where $c_{0}\in R.$ Thus, by the boundary condition $\omega \left(a\right)=\eta_{1}$, we get

$$\begin{array}{c} c_{0}=\eta_{1}+\frac{\left(1-\vartheta_{2}\right)}{\Lambda (\vartheta_{2})}[\frac{\eta_{1}\gamma \left(a\right)}{\mu (a)}-\frac{\xi_{0}}{\mu (a)}]. \end{array}$$ (20)

Therefore, by substituting the value of *c*_0_ into Eq (19), one have

ω(υ)=η1+(1-ϑ2)Λ(ϑ2)[η1γ(a)μ(a)-ξ0μ(a)]-IABaϑ2,ρ[γ(υ)μ(υ)ω(υ)]+IABaϑ2,ρ[1μ(υ)∑k=0mξkk!(ρ^a(υ))k]+IABaϑ2,ρ[1μ(υ)IABaϑ1,ρq(υ)]. (21)

Hence, the proof is finished.

As a consequence of Lemma 3.1, we conclude the following interesting lemma.

**Lemma 3.2**. *Let* *ϑ*_1_ ∈ (*m*, *m* + 1], *ϑ*_2_ ∈ (0, 1] *and* *ω* ∈ *χ*. *Then, the equivalent solution form of the* ρ-ABC FGLSLP (3), *is*

ω(υ)=η1+(1-ϑ2)Λ(ϑ2)[η1γ(a)μ(a)-ξ0μ(a)]-IABaϑ2,ρ[γ(υ)μ(υ)ω(υ)]+IABaϑ2,ρ[1μ(υ)∑k=0mξkk!(ρ^a(υ))k]+IABaϑ2,ρ[1μ(υ)IABaϑ1,ρg(υ,ω(υ))]. (22)

In this position, we need to give the following hypotheses:

**(G1)** g(υ,ω(υ))∈C(J×R,R) ;
**(G2)** There is a positive function *α* with bounds ‖*α*‖, such that
  $$\begin{array}{c} |g(\upsilon ,\omega (\upsilon ))-g(\upsilon ,\varpi (\upsilon ))|\leq \left(\upsilon \right)\|\omega -\varpi \|,\;\forall \upsilon \in J,\;\omega ,\varpi \in \chi ; \end{array}$$ (23)
**(G3)** There exists a constant *φ* ≥ 0, with |g(υ,ω)|≤φ‖ω‖.

Now, we investigate the uniqueness results by helping Banach contraction theorem.

**Theorem 3.3**. *Let the hypotheses* (G1)–(G2) *fulfilled. If*

$$\begin{array}{c} \Delta ≔\frac{\gamma^{*}{\hat{\Lambda }}_{1}+\left\|\alpha \right\|\;{\hat{\Lambda }}_{2}}{\mu_{*}}<1, \end{array}$$ (24)

*then there is an exactly one solution of the* FGLSLP (3) *on* J, *where* μ_*_ = inf_*υ*∈J_ |*μ*(*υ*)|, γ* = sup_*υ*∈J_ |*γ*(*υ*)|, *and*

$$\begin{array}{cc} {\hat{\Lambda }}_{1} & ≔\frac{1-\vartheta_{2}}{\left|\Lambda (\vartheta_{2})\right|}+\frac{\vartheta_{2}{\left({\hat{\rho }}_{a}\left(\upsilon \right)\right)}^{\vartheta_{2}}}{|\Lambda \left(\vartheta_{2}\right)|\;\Gamma \left(\vartheta_{2}+1\right)},\\ {\hat{\Lambda }}_{2} & ≔(\frac{\left(1-\vartheta_{2}\right)}{\Lambda (\vartheta_{2})}+\frac{\left(\vartheta_{2}\right){\left({\hat{\rho }}_{a}\left(\upsilon \right)\right)}^{\vartheta_{2}}}{\Lambda \left(\vartheta_{2}\right)\Gamma \left(\vartheta_{2}+1\right)})\cdot (\frac{\left(m+1-\vartheta_{1}\right){\left({\hat{\rho }}_{a}\left(\upsilon \right)\right)}^{m}}{\Lambda \left(\vartheta_{1}-m\right)\Gamma \left(m+1\right)}+\frac{\left(\vartheta_{1}-m\right){\left({\hat{\rho }}_{a}\left(\upsilon \right)\right)}^{\vartheta_{1}}}{\Lambda \left(\vartheta_{1}-m\right)\Gamma \left(\vartheta_{1}+1\right)}). \end{array}$$ (25)

*Proof*. The idea of the proof is transform the FGLSLP (3) into a fixed point of the operator K:χ→χ given by

K(ω)(υ)=η1+(1-ϑ2)Λ(ϑ2)[η1γ(a)μ(a)-ξ0μ(a)]-IABaϑ2,ρ[γ(υ)μ(υ)ω(υ)]+IABaϑ2,ρ[1μ(υ)∑k=0mξkk!(ρ^a(υ))k]+IABaϑ2,ρ[1μ(υ)IABaϑ1,ρg(υ,ω(υ))]. (26)

Notice that K is well defined. Let *B*_*r*_ = {*ω* ∈ *χ* : ‖*ω*‖ ≤ *r*} be a closed, convex and bounded subset of *χ*, where the fixed constant *r* satisfies

$$\begin{array}{c} r[1-(\frac{\gamma^{*}}{\mu_{*}}{\hat{\Lambda }}_{1}+\frac{\left\|\alpha \right\|}{\mu_{*}}{\hat{\Lambda }}_{2})]\geq \frac{g_{0}}{\mu_{*}}{\hat{\Lambda }}_{2}+\frac{1}{\mu_{*}}{\hat{\Lambda }}_{3}+{\hat{\Lambda }}_{4}, \end{array}$$ (27)

where $g_{0}=\text{sup}\;\left\{g\left(\upsilon ,0\right)\;:\;\upsilon \in J\right\}$, and

$$\begin{array}{cc} {\hat{\Lambda }}_{3} & ≔\sum_{k=0}^{m}\frac{|\xi_{k}|}{k!}(\frac{\left(1-\vartheta_{2}\right){\left({\hat{\rho }}_{a}\left(\upsilon \right)\right)}^{k}}{\left|\Lambda (\vartheta_{2})\right|}+\frac{\vartheta_{2}\Gamma \left(k+1\right){\left({\hat{\rho }}_{a}\left(\upsilon \right)\right)}^{k+\vartheta_{2}}}{|\Lambda \left(\vartheta_{2}\right)|\;\Gamma \left(\vartheta_{2}+k+1\right)}),\\ {\hat{\Lambda }}_{4} & ≔|\eta_{1}|+\frac{|1-\vartheta_{2}|}{\left|\Lambda (\vartheta_{2})\right|}[\frac{|\eta_{1}\left|\;|\gamma \left(a\right)\right|}{\left|\mu (a)\right|}+\frac{|\xi_{0}|}{\left|\mu (a)\right|}]. \end{array}$$ (28)

Next, we prove that $KB_{r}\subset B_{r}$ and by using the triangle inequality

$$\begin{array}{c} |g(\upsilon ,\omega (\upsilon ))|\leq |g(\upsilon ,\omega (\upsilon ))-g\left(\upsilon ,0\right)|+|g(\upsilon ,0)|, \end{array}$$ (29)

we have

|K(ω)(υ)|≤|η1+(1-ϑ2)Λ(ϑ2)[η1γ(a)μ(a)-ξ0μ(a)]-IABaϑ2,ρ[γ(υ)μ(υ)ω(υ)]+IABaϑ2,ρ[1μ(υ)∑k=0mξkk!(ρ^a(υ))k]+IABaϑ2,ρ[1μ(υ)IABaϑ1,ρg(υ,ω(υ))]|≤|η1|+|1-ϑ2||Λ(ϑ2)|[|η1||γ(a)||μ(a)|+|ξ0||μ(a)|]+IABaϑ2,ρ[γ*μ*|ω(υ)|]+IABaϑ2,ρ[1μ*|∑k=0mξkk!(ρ^a(υ))k|]+IABaϑ2,ρ[1μ*IABaϑ1,ρ(|g(υ,ω(υ))-g(υ,0)|+|g(υ,0)|)]≤|η1|+|1-ϑ2||Λ(ϑ2)|[|η1||γ(a)||μ(a)|+|ξ0||μ(a)|]+γ*rμ*((1-ϑ2)|Λ(ϑ2)|+ϑ2(ρ^a(υ))ϑ2|Λ(ϑ2)|Γ(ϑ2+1))+1μ*∑k=0m|ξk|k!((1-ϑ2)Γ(k+1)(ρ^a(υ))k|Λ(ϑ2)|Γ(k+1)+ϑ2Γ(k+1)(ρ^a(υ))k+ϑ2|Λ(ϑ2)|Γ(ϑ2+k+1))+‖α‖r+g0μ*((1-ϑ2)Λ(ϑ2)+(ϑ2)(ρ^a(υ))ϑ2Λ(ϑ2)Γ(ϑ2+1))×((m+1-ϑ1)(ρ^a(υ))mΛ(ϑ1-m)Γ(m+1)+(ϑ1-m)(ρ^a(υ))ϑ1Λ(ϑ1-m)Γ(ϑ1+1))≤(γ*μ*Λ^1+‖α‖μ*Λ^2)r+g0μ*Λ^2+1μ*Λ^3+Λ^4≤r.

In the following, we go to prove that K is a contraction mapping. Let $\omega ,\hat{\omega }\in \chi$, for *υ* ∈ J, and using (G2), it follows that

|K(ω)(υ)-K(ω^)(υ)|≤IABaϑ2,ρ[γ(υ)μ(υ)|ω(υ)-ω^(υ)|]+IABaϑ2,ρ[1μ(υ)IABaϑ1,ρ|g(υ,ω(υ))-g(υ,ω^(υ))|]≤‖ω-ω^‖IABaϑ2,ρ[γ(υ)μ(υ)(1)(υ)]+‖α‖‖ω-ω^‖IABaϑ2,ρ[1μ(υ)IABaϑ1,ρ(1)(υ)]≤γ*μ*‖ω-ω^‖((1-ϑ2)|Λ(ϑ2)|+ϑ2(ρ^a(υ))ϑ2|Λ(ϑ2)|Γ(ϑ2+1))+‖α‖μ*‖ω-ω^‖((m+2-ϑ1-ϑ2)(ρ^a(υ))m+1|Λ(ϑ1+ϑ2-m)|Γ(m+2)+(ϑ1+ϑ2-m)(ρ^a(υ))ϑ1+ϑ2|Λ(ϑ1+ϑ2-m)|Γ(ϑ1+ϑ2+1))≤‖ω-ω^‖[γ*μ*((1-ϑ2)|Λ(ϑ2)|+ϑ2(ρ^a(υ))ϑ2|Λ(ϑ2)|Γ(ϑ2+1))+‖α‖μ*((1-ϑ2)Λ(ϑ2)+(ϑ2)(ρ^a(υ))ϑ2Λ(ϑ2)Γ(ϑ2+1))·((m+1-ϑ1)(ρ^a(υ))mΛ(ϑ1-m)Γ(m+1)+(ϑ1-m)(ρ^a(υ))ϑ1Λ(ϑ1-m)Γ(ϑ1+1))]≤(γ*μ*Λ^1+‖α‖μ*Λ^2)‖ω-ω^‖,

which implies $\|K\left(\omega \right)-K\left(\hat{\omega }\right)\|\leq \Delta \|\omega -\hat{\omega }\|$. In view of Δ < 1, we have K is a contraction. Therefore, by Banach FPT the K possesses an exactly one fixed point and acts a solution of the FGLSLP (3).

In follows, we discuss the existence result by utilizing Krasnoselskii’s FPT.

**Theorem 3.4**. *let the hypotheses* (G1)–(G3) *fulfilled. Then*, FGLSLP (3) *possesses at least one solution, on condition of*

$$\begin{array}{c} \gamma^{*}{\hat{\Lambda }}_{1}<\mu_{*}. \end{array}$$ (30)

*Proof*. According to the hypothesis (G3), we set

$$\begin{array}{c} P[1-(\frac{\gamma^{*}}{\mu_{*}}{\hat{\Lambda }}_{1}+\frac{\left\|\alpha \right\|}{\mu_{*}}{\hat{\Lambda }}_{2})]\geq \frac{1}{\mu_{*}}{\hat{\Lambda }}_{3}+{\hat{\Lambda }}_{4}, \end{array}$$ (31)

where *B*_P_ = {*ω* ∈ *χ* : ‖*ω*‖ ≤ P}. By dividing the mapping K:χ→χ given by Eq (26) as $K=K_{1}+K_{2},$ where $K_{1}$, $K_{2}$ are given by

K1(ω)(υ)=η1+(1-ϑ2)Λ(ϑ2)[η1γ(a)μ(a)-ξ0μ(a)]-IABaϑ2,ρ[γ(υ)μ(υ)ω(υ)]+IABaϑ2,ρ[1μ(υ)∑k=0mξkk!(ρ^a(υ))k],K2(ω)(υ)≔IABaϑ2,ρ[1μ(υ)IABaϑ1,ρg(υ,ω(υ))], (32)

for *υ* ∈ J. The producers of our proof will be divide into several steps:

**Step 1:** $K_{1}\left(\omega \right)+K_{2}\left(\omega \right)\in B_{P}$ . Let *ω* ∈ *B*_P_ and *υ* ∈ J. Then

|K1(ω)(υ)+K2(ω)(υ)|≤|η1+(1-ϑ2)Λ(ϑ2)[η1γ(a)μ(a)-ξ0μ(a)]-IABaϑ2,ρ[γ(υ)μ(υ)ω(υ)]+IABaϑ2,ρ[1μ(υ)∑k=0mξkk!(ρ^a(υ))k]+IABaϑ2,ρ[1μ(υ)IABaϑ1,ρg(υ,ω(υ))]|≤|η1|+|1-ϑ2||Λ(ϑ2)|[|η1||γ(a)||μ(a)|+|ξ0||μ(a)|]+IABaϑ2,ρ[γ*μ*|ω(υ)|]+IABaϑ2,ρ[1μ*|∑k=0mξkk!(ρ^a(υ))k|]+IABaϑ2,ρ[1μ*IABaϑ1,ρ|g(υ,ω(υ))|]≤|η1|+|1-ϑ2||Λ(ϑ2)|[|η1||γ(a)||μ(a)|+|ξ0||μ(a)|]+γ*Pμ*((1-ϑ2)|Λ(ϑ2)|+ϑ2(ρ^a(υ))ϑ2|Λ(ϑ2)|Γ(ϑ2+1))+1μ*∑k=0m|ξk|k!((1-ϑ2)Γ(k+1)(ρ^a(υ))k|Λ(ϑ2)|Γ(k+1)+ϑ2Γ(k+1)(ρ^a(υ))k+ϑ2|Λ(ϑ2)|Γ(ϑ2+k+1))+φPμ*((1-ϑ2)Λ(ϑ2)+(ϑ2)(ρ^a(υ))ϑ2Λ(ϑ2)Γ(ϑ2+1))·((m+1-ϑ1)(ρ^a(υ))mΛ(ϑ1-m)Γ(m+1)+(ϑ1-m)(ρ^a(υ))ϑ1Λ(ϑ1-m)Γ(ϑ1+1))≤(γ*μ*Λ^1+φμ*Λ^2)P+1μ*Λ^3+Λ^4≤P.

Hence, $\|K_{1}\left(\omega \right)+K_{2}\left(\omega \right)\|\leq P$, which shows that $K_{1}\omega +K_{2}\omega \in B_{P}$.

**Step 2:** $K_{1}$ is a contration mapping on *B*_P_. The mapping $K_{1}$ is a contraction on condition of $\gamma^{*}{\hat{\Lambda }}_{1}<\mu_{*}$, according to the contractility of K as in Theorem 3.3.

**Step 3:** $K_{2}$ is completely continuous on *B*_P_. By continuity of g(·,ω(·)), it implies that $K_{2}$ is continuous. For *ω* ∈ *B*_P_

$$\begin{array}{c} \|K_{2}\omega \|=\underset{\upsilon \in J}{\text{sup}}\;|K_{2}\omega \left(\upsilon \right)|\leq \frac{\varphi \|\omega \|}{\mu_{*}}(\frac{\left(1-\vartheta_{2}\right)}{\Lambda (\vartheta_{2})}+\frac{\left(\vartheta_{2}\right){\left({\hat{\rho }}_{a}\left(\upsilon \right)\right)}^{\vartheta_{2}}}{\Lambda \left(\vartheta_{2}\right)\Gamma \left(\vartheta_{2}+1\right)}) \end{array}$$ (33)

$$\begin{array}{cc} & \;\times (\frac{\left(m+1-\vartheta_{1}\right){\left({\hat{\rho }}_{a}\left(\upsilon \right)\right)}^{m}}{\Lambda \left(\vartheta_{1}-m\right)\Gamma \left(m+1\right)}+\frac{\left(\vartheta_{1}-m\right){\left({\hat{\rho }}_{a}\left(\upsilon \right)\right)}^{\vartheta_{1}}}{\Lambda \left(\vartheta_{1}-m\right)\Gamma \left(\vartheta_{1}+1\right)})≔\frac{\varphi }{\mu_{*}}P{\hat{\Lambda }}_{2},\;\omega \in B_{P}. \end{array}$$ (34)

We get $\|K_{2}\omega \|\leq \frac{\varphi }{\mu_{*}}P{\hat{\Lambda }}_{2}$, which yields that $K_{2}$ is uniformly bounded on *B*_P_.

**Step 4:** The mapping $K_{2}$ is compactness. For *ω* ∈ *B*_P_ and *υ* ∈ J, we can evaluate the mapping derivative as below:

|(K2ω)ρ(1)(υ)|≤(1ρ′(υ)ddυ)(IABaϑ2,ρ[1μ(υ)IABaϑ1,ρ|g(υ,ω(υ))|])≤φPμ*(1ρ′(υ)ddυ)((1-ϑ2)Λ(ϑ2)+(ϑ2)(ρ^a(υ))ϑ2Λ(ϑ2)Γ(ϑ2+1))×((m+1-ϑ1)(ρ^a(υ))mΛ(ϑ1-m)Γ(m+1)+(ϑ1-m)(ρ^a(υ))ϑ1Λ(ϑ1-m)Γ(ϑ1+1))≔ℓ, (35)

where we used the fact DρkIRLam,ρ=IRLam-k,ρ and use definition $\omega_{\rho }^{\left(k\right)}\left(\upsilon \right)$ from Eq (9) for *k* = 0, 1, …, *m* − 1. Hence, for each *υ*_1_, *υ*_2_ ∈ J with $a<\upsilon_{1}<\upsilon_{2}<b$ and for *ω* ∈ *B*_P_, we get

$$\begin{array}{c} |(K_{2}\omega )\left(\upsilon_{2}\right)-(K_{2}\omega )\left(\upsilon_{1}\right)|=\int_{\upsilon_{1}}^{\upsilon_{2}}\rho^{\prime }\left(s\right)|{\left(K_{2}\omega \right)}_{\rho }^{\left(1\right)}\left(s\right)|ds\leq \ell {\hat{\rho }}_{\upsilon_{1}}\left(\upsilon_{2}\right), \end{array}$$ (36)

which tends to zero independent of *ω* as *υ*_2_ → *υ*_1_. So, $K_{2}$ is equicontinuous. In view of the Arzelà–Ascoli theorem, $K_{2}$ is compact mapping on $B_{P}$. Therefore, the conditions of the Krasnoselskii’s FPT 2.6 satisfy, thus there is at least one solution of FGLSLP (3) on J.

## 4 Stability criteria

In this section, the stability analysis in the sense of the UH and UHR are established with their generalized form for solutions of the ρ-ABC FGLSLP (3). For more details about the following definitions, we refer the readers to these works [20, 21].

**Definition 4.1**. *The* ρ-ABC FGLSLP (3) *is*

1. UH *stable if there is* $0<\sigma_{g}\in R$ *where, for all ϵ* > 0 and for every ω*(*υ*) ∈ *χ* *satisfying*
  |DABCaϑ1,ρ[μ(υ)DABCaϑ2,ρ+γ(υ)]ω*(υ)-g(υ,ω*(υ))|<ϵ, (37)
  *there is ω*(*υ*) ∈ *χ* *satisfying the* FGLSLP (3) *with* $|\omega^{*}\left(\upsilon \right)-\omega \left(\upsilon \right)|\leq \epsilon \sigma_{g}$, *for each* *υ* ∈ J;
2. *Generalized* UH *stable if there is* $\sigma_{g}\in C(R^{+},R^{+})$ *with* *σ*_g_(0) = 0 *where, for all ϵ* > 0 and for every ω*(*υ*) ∈ *χ* *satisfying* (37), *then there is ω*(*υ*) ∈ *χ* *satisfying the system* (3) *with* $|\omega^{*}\left(\upsilon \right)-\omega \left(\upsilon \right)|\leq \sigma_{g}\left(\epsilon \right)$.
3. UHR *stable if there are* ϕ∈C(J,R), *and* $0<\sigma_{g,\phi }\in R$, *where, for all* *ϵ* > 0 and for every ω*(*υ*) ∈ *χ* *satisfying*
  |DABCaϑ1,ρ[μ(υ)DABCaϑ2,ρ+γ(υ)]ω*(υ)-g(υ,ω*(υ))|<ϵϕ(υ), (38)
  *there is* *ω*(*υ*) ∈ *χ* *satisfying the* FGLSLP (3) *with* $|\omega^{*}\left(\upsilon \right)-\omega \left(\upsilon \right)|\leq \sigma_{g,\phi }\epsilon \phi \left(\upsilon \right)$, *for each υ* ∈ J;
4. *Generalized* UHR *stable if there are* ϕ∈C(J,R) , *and* $0<\sigma_{g,\phi }\in R$, *with* *σ*_g_(0) = 0, *and for every* ω*(*υ*) ∈ *χ* *satisfying* (38), *then there is* *ω*(*υ*) ∈ *χ* *satisfying the system* (3) *with* $|\omega^{*}\left(\upsilon \right)-\omega \left(\upsilon \right)|\leq \sigma_{g,\phi }\;\phi \left(\upsilon \right)$.

**Remark 4.2**. *We note the following*:

1. *If* ω*(*υ*) ∈ *χ* *is a solution for* (37) *if and only if there is* *σ* ∈ *χ* *depending on* ω* *such that, for each υ* ∈ J, |*σ*(*υ*)| < *ϵ* *and*
  DABCaϑ1,ρ[μ(υ)DABCaϑ2,ρ+γ(υ)]ω*(υ)=g(υ,ω*(υ))+σ(υ). (39)
2. *If* ω*(*υ*) ∈ *χ* *is a solution for* (38) *if and only if there is* ς∈χ *depending on* ω* *such that, for each υ* ∈ J, |ς(υ)|<ϵϕ(υ) *and*
  DABCaϑ1,ρ[μ(υ)DABCaϑ2,ρ+γ(υ)]ω*(υ)=g(υ,ω*(υ))+ς(υ). (40)
3. *There are real number Z*_*ϕ*_ > 0, *and increasing function ϕ*(*υ*) ∈ *χ* *such that* IABaϑ2,ρ[1μ(υ)IABaϑ1,ρϕ(υ)]≤Zϕϕ(υ),∀υ∈J.

**Theorem 4.3**. *Let the hypotheses* (G1)–(G2) *and* Δ < 1 *are hold. Then, the solution of* FGLSLP (3) *is* UH *and generalized* UH *stable*.

*Proof*. Let *ϵ* > 0, *and* $\overset{ˇ}{\omega }\in \chi$ *be a function which verifying the inequality* (37) *and consider ω* ∈ *χ* *the unique solution of the problem*

DABCaϑ1,ρ[μ(υ)DABCaϑ2,ρ+γ(υ)]ω(υ)=g(υ,ω(υ)),∀υ∈J, (41)

with boundary conditions in (3). Now, Lemma 3.2 implies that

ω(υ)=η1+1-ϑ2Λ(ϑ2)[η1γ(a)μ(a)-ξ0μ(a)]-IABaϑ2,ρ[γ(υ)μ(υ)ω(υ)]+IABaϑ2,ρ[1μ(υ)∑k=0mξkk!(ρ^a(υ))k]+IABaϑ2,ρ[1μ(υ)IABaϑ1,ρg(υ,ω(υ))]. (42)

Since $\overset{˘}{\omega }$ verifying (37). Thus by Remark 4.2, we have

DABCaϑ1,ρ[μ(υ)DABCaϑ2,ρ+γ(υ)]ω˘(υ)=g(υ,ω˘(υ))+σ(υ), (43)

with the boundary conditions:

{ω˘(a)=η1,[μ(a)DABCaϑ2,ρ+γ(a)]ρ(k)ω˘(a)=ξk,0≤k≤m. (44)

Again, Lemma 3.2 implies that

ω˘(υ)=η1+1-ϑ2Λ(ϑ2)[η1γ(a)μ(a)-ξ0μ(a)]-IABaϑ2,ρ[γ(υ)μ(υ)ω˘(υ)]+IABaϑ2,ρ[1μ(υ)∑k=0mξkk!(ρ^a(υ))k]+IABaϑ2,ρ[1μ(υ)IABaϑ1,ρg(υ,ω˘(υ))]+IABaϑ2,ρ[1μ(υ)IABaϑ1,ρσ(υ)]. (45)

Now, for each *υ* ∈ J, we find

|ω˘(υ)-ω(υ)|≤|ω˘(υ)-η1+1-ϑ2Λ(ϑ2)[η1γ(a)μ(a)-ξ0μ(a)]-IABaϑ2,ρ[γ(υ)μ(υ)ω(υ)]+IABaϑ2,ρ[1μ(υ)∑k=0mξkk!(ρ^a(υ))k]+IABaϑ2,ρ[1μ(υ)IABaϑ1,ρg(υ,ω(υ))]|. (46)

Hence, by using the Remark 4.2 and (G2) we can get $\|\overset{˘}{\omega }-\omega \|\leq \Theta \epsilon +\Delta \|\overset{˘}{\omega }-\omega \|$, where Δ is defined in (24) and Θ given by

$$\begin{array}{c} \Theta ≔\frac{1}{\mu^{*}}\left(\frac{\left(1-\vartheta_{2}\right)}{\Lambda \left(\vartheta_{2}\right)}+\frac{\left(\vartheta_{2}\right){\left({\hat{\rho }}_{a}\left(\upsilon \right)\right)}^{\vartheta_{2}}}{\Lambda \left(\vartheta_{2}\right)\Gamma \left(\vartheta_{2}+1\right)}\right)\cdot \left(\frac{\left(m+1-\vartheta_{1}\right){\left({\hat{\rho }}_{a}\left(\upsilon \right)\right)}^{m}}{\Lambda \left(\vartheta_{1}-m\right)\Gamma \left(m+1\right)}+\frac{\left(\vartheta_{1}-m\right){\left({\hat{\rho }}_{a}\left(\upsilon \right)\right)}^{\vartheta_{1}}}{\Lambda \left(\vartheta_{1}-m\right)\Gamma \left(\vartheta_{1}+1\right)}\right). \end{array}$$ (47)

In consequence, it follows that $\|\overset{˘}{\omega }-\omega \|\leq \frac{1}{1-\Delta }\Theta \epsilon$. Therefor, the UH stability condition is satisfied whenever $\sigma_{g}=\frac{\Theta }{1-\Delta }$. More generally, for $\sigma_{g}\left(\epsilon \right)=\frac{\Theta }{1-\Delta }\epsilon$, $\sigma_{g}\left(0\right)=0$, the condition of generalized UH stability is also satisfied. Thus the proof is completed.

**Theorem 4.4**. *Let the hypotheses* (G1)–(G2) *and* Δ < 1 *are hold. Then, the solution of* FGLSLP (3) *is* UHR *and generalized* UHR *stable*.

*Proof*. Let *ϵ* > 0, and $\overset{ˇ}{\omega }\in \chi$ be a function which verifying the inequality (38) and consider *ω* ∈ *χ* the unique solution of the problem

DABCaϑ1,ρ[μ(υ)DABCaϑ2,ρ+γ(υ)]ω(υ)=g(υ,ω(υ)),∀υ∈J, (48)

with boundary conditions in (3). Now, Lemma 3.2 implies that

ω(υ)=η1+1-ϑ2Λ(ϑ2)[η1γ(a)μ(a)-ξ0μ(a)]-IABaϑ2,ρ[γ(υ)μ(υ)ω(υ)]+IABaϑ2,ρ[1μ(υ)∑k=0mξkk!(ρ^a(υ))k]+IABaϑ2,ρ[1μ(υ)IABaϑ1,ρg(υ,ω(υ))]. (49)

Since $\overset{˘}{\omega }$ verifying (38). Thus by Remark 4.2, we have

DABCaϑ1,ρ[μ(υ)DABCaϑ2,ρ+γ(υ)]ω˘(υ)=g(υ,ω˘(υ))+ς(υ), (50)

with the boundary conditions:

{ω˘(a)=η1,[μ(a)DABCaϑ2,ρ+γ(a)]ρ(k)ω˘(a)=ξk,0≤k≤m. (51)

Then, by Lemma 3.2, we get

ω˘(υ)=η1+1-ϑ2Λ(ϑ2)[η1γ(a)μ(a)-ξ0μ(a)]-IABaϑ2,ρ[γ(υ)μ(υ)ω˘(υ)]+IABaϑ2,ρ[1μ(υ)∑k=0mξkk!(ρ^a(υ))k]+IABaϑ2,ρ[1μ(υ)IABaϑ1,ρg(υ,ω˘(υ))]+IABaϑ2,ρ[1μ(υ)IABaϑ1,ρς(υ)]. (52)

Now, for each *υ* ∈ J, we find

|ω˘(υ)-ω(υ)|≤|ω˘(υ)-η1+1-ϑ2Λ(ϑ2)[η1γ(a)μ(a)-ξ0μ(a)]-IABaϑ2,ρ[γ(υ)μ(υ)ω(υ)]+IABaϑ2,ρ[1μ(υ)∑k=0mξkk!(ρ^a(υ))k]+IABaϑ2,ρ[1μ(υ)IABaϑ1,ρg(υ,ω(υ))]|. (53)

Thus, by using the Remark 4.2 and (G2) we can get $\|\overset{˘}{\omega }-\omega \|\leq \epsilon Z_{\phi }\phi \left(\upsilon \right)+\Delta \left\|\overset{˘}{\omega }-\omega \right\|$, where Δ is defined in (24).

In consequence, it follows that $\|\overset{˘}{\omega }-\omega \|\leq \frac{1}{1-\Delta }\epsilon Z_{\phi }\phi \left(\upsilon \right)$. Hence, the UHR stability condition is satisfied whenever $\sigma_{g,\phi }=\frac{Z_{\phi }}{1-\Delta }$. More generally, for *ϵ* = 1, the condition of generalized UHR stability is also satisfied. Thus the proof is completed.

## 5 Numerical examples with discussion

Here, we present different version of the function *ρ*, and various modes *ϑ*_1_, *ϑ*_2_.

**Example 5.1**. *Based on the problem* (3), *we consider the following* ρ-ABC FGLSLP *on domain* J = [1, 2.25], *with* a=1, b=2.25 *and m* = 3 *as form*

DABCaϑ1,eυ/2[1υDABCa2/3,eυ/2+135υcos(πυ)]ω(υ)=3tan-1υ(sinω(υ)+63)5(2+|υ|), (54)

*when*

$$\begin{array}{c} \vartheta_{1}\in \{\frac{10}{3},\;\frac{7}{2},\;\frac{39}{10}\}\subset \left(3,4\right], \end{array}$$ (55)

*under conditions* $\omega \left(1\right)=-\frac{14}{\sqrt{129}}$ *and*

[DABC12/3,e1/2-135]e1/2(0)ω(1)=-419,[DABC12/3,e1/2-135]e1/2(1)ω(1)=713,[DABC12/3,e1/2-135]e1/2(2)ω(1)=-1633,[DABC12/3,e1/2-135]e1/2(3)ω(1)=289. (56)

*Clearly*, $\vartheta_{2}=\frac{2}{3}\in \left(0,1\right]$, $\eta_{1}=\frac{-14}{\sqrt{129}}$, *m* = 3, $\xi_{0}=\frac{-4}{19}$, $\xi_{1}=\frac{7}{13}$, $\xi_{2}=\frac{-16}{33}$, $\xi_{3}=\frac{28}{9}$, $\xi_{4}=\frac{28}{9}$ *and* $\rho \left(\upsilon \right)=e^{\upsilon /2}\in C^{3}\left(\left(1,\;2.25\right),R^{+}\right)$. *We define* $\mu \left(\upsilon \right)=\frac{1}{\upsilon }\in C\left(J,R\backslash \left\{0\right\}\right)$, $\gamma \left(\upsilon \right)=\frac{1}{35}\upsilon \;\text{cos}\;\left(\pi \upsilon \right)\in \chi$,

$$\begin{array}{c} g(\upsilon ,\omega (\upsilon ))=\frac{3\;{\text{tan}}^{-1}\;\upsilon (\text{sin}\;\omega \left(\upsilon \right)+\sqrt[{3}]{6})}{\sqrt{5}\left(2+|\upsilon |\right)}, \end{array}$$ (57)

*and consider the normalization function* $\Lambda (\upsilon )=e^{\upsilon^{2}-\upsilon }$. *There is no doubt about the continuity of function* g(υ,ω(υ)), *this means condition* (G1) *is satisfied. In addition, for* $\omega ,\hat{\omega }\in \chi$, *we have*

$$\begin{array}{cc} \left|g(\upsilon ,\omega (\upsilon )\right) & -g(\upsilon ,\hat{\omega }\left(\upsilon \right))|\\ & =|\frac{3\;{\text{tan}}^{-1}\;\upsilon (\text{sin}\;\omega \left(\upsilon \right)+\sqrt[{3}]{6})}{\sqrt{5}\left(2+|\upsilon |\right)}-\frac{3\;{\text{tan}}^{-1}\;\upsilon (\text{sin}\;\hat{\omega }\left(\upsilon \right)+\sqrt[{3}]{6})}{\sqrt{5}\left(2+|\upsilon |\right)}|\\ & =\frac{3\;{\text{tan}}^{-1}\;\upsilon }{\sqrt{5}\left(2+|\upsilon |\right)}|\text{sin}\;\omega \left(\upsilon \right)-\text{sin}\;\hat{\omega }\left(\upsilon \right)|\leq \left\|\alpha \left(\upsilon \right)\right\||\omega \left(\upsilon \right)-\hat{\omega }\left(\upsilon \right)|, \end{array}$$

*where* $\alpha (\upsilon )=\frac{3}{\sqrt{5}\left(2+|\upsilon |\right)}\;{\text{tan}}^{-1}\;\upsilon$ *with* ‖*α*‖ ≃ 0.5154. *Indeed, the correctness of assumption* (G2) *can be accepted. Also, by considering the* $\varphi ≔\frac{1}{\sqrt{5}}\left(1+\sqrt[{3}]{6}\right)\;{\text{tan}}^{-1}\;\left(2.25\right)$, *hypothesis* (G3) *holds*:

$$\begin{array}{c} |g(\upsilon ,\omega (\upsilon ))|=|\frac{3\;{\text{tan}}^{-1}\;\upsilon (\text{sin}\;\omega \left(\upsilon \right)+\sqrt[{3}]{6})}{\sqrt{5}\left(2+|\upsilon |\right)}|\leq \varphi \left\|\omega \left(\upsilon \right)\right\|, \end{array}$$ (58)

*and*

$$\begin{array}{c} g_{0}=\text{sup}\;\{g(\upsilon ,0)\;:\;\upsilon \in J\}=\underset{\upsilon \in J}{\text{sup}}\;\frac{3\sqrt[{3}]{6}\;{\text{tan}}^{-1}\;\upsilon }{\sqrt{5}\left(2+|\upsilon |\right)}=\frac{\sqrt[{3}]{6}}{\sqrt{5}}\;{\text{tan}}^{-1}\;\left(2.25\right). \end{array}$$ (59)

*Now, by using* Eq (25), *we obtain*

$$\begin{array}{cc} {\hat{\Lambda }}_{1} & =\frac{1-\vartheta_{2}}{\left|\Lambda (\vartheta_{2})\right|}+\frac{\vartheta_{2}{\left({\hat{\rho }}_{a}\left(\upsilon \right)\right)}^{\vartheta_{2}}}{|\Lambda \left(\vartheta_{2}\right)|\;\Gamma \left(\vartheta_{2}+1\right)}\\ & =\frac{{\left(e^{2.25}-e\right)}^{3}}{3|\text{exp}\;({\left(\frac{2}{3}\right)}^{2}-\frac{2}{3})|}+\frac{2{\left(e^{2.25}-e\right)}^{2/3}}{3|\text{exp}\;({\left(\frac{2}{3}\right)}^{2}-\frac{2}{3})|\Gamma (\frac{5}{3})}≃1.5877, \end{array}$$ (60)

$$\begin{array}{cc} {\hat{\Lambda }}_{2} & ≃\{\begin{array}{cc} 0.3499, & \vartheta_{1}=\frac{10}{3},\\ 0.2584, & \vartheta_{1}=\frac{7}{2},\\ 0.0881, & \vartheta_{1}=\frac{39}{10}, \end{array} \end{array}$$ (61)

*and by employing* Eq (24) *we get*

$$\begin{array}{c} \Delta =\frac{\gamma^{*}\;{\hat{\Lambda }}_{1}+\left\|\alpha \right\|\;{\hat{\Lambda }}_{2}}{\mu_{*}}≃\{\begin{array}{cc} 0.5682, & \vartheta_{1}=\frac{10}{3},\\ 0.4621, & \vartheta_{1}=\frac{7}{2},\\ 0.2646, & \vartheta_{1}=\frac{39}{10}, \end{array}\}<1. \end{array}$$ (62)

**Fig 1:**
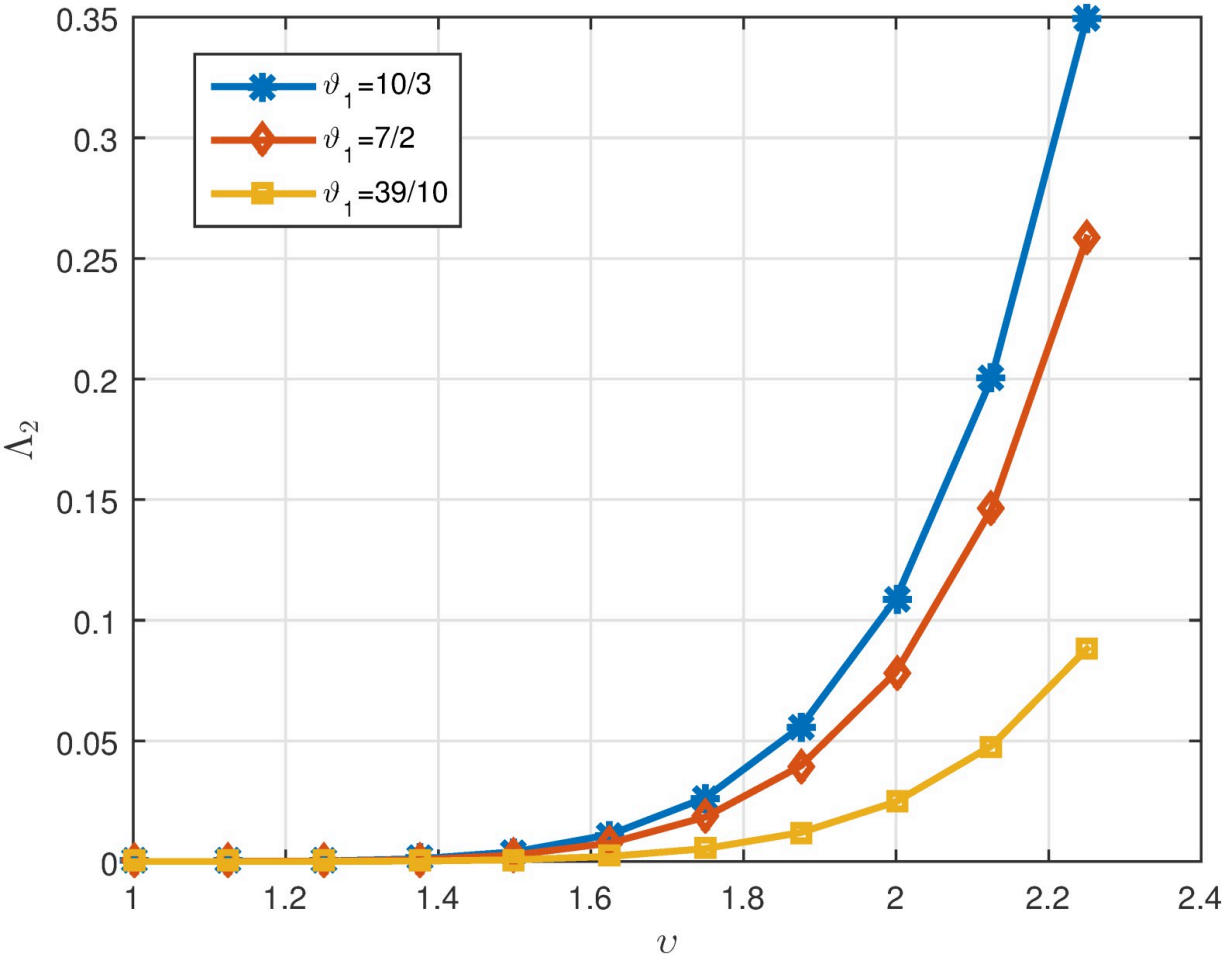
2D plots of ${\hat{\Lambda }}_{2}$ for three cases of *ϑ*_1_ when *υ* ∈ J in Example 5.1.

Table 1 *present the numeric values of* ${\hat{\Lambda }}_{1}$ , ${\hat{\Lambda }}_{2}$ *and* Δ *for υ* ∈ [1, 2.25]. *The values of* ${\hat{\Lambda }}_{2}$ *and* Δ *are also shown in* Figs 1 *and* 2 *for three cases of* *ϑ*_1_, *respectively. The variable* ${\hat{\Lambda }}_{1}$ *is not dependent on* *ϑ*_1_. *Therefore, conditions of Theorem 3.3 are hold for all cases of order* *ϑ*_1_. *Therefore*, ρ-ABC FGLSLP (54) *admits unique solution on domain* J = [1, 2.25].

**Fig 2:**
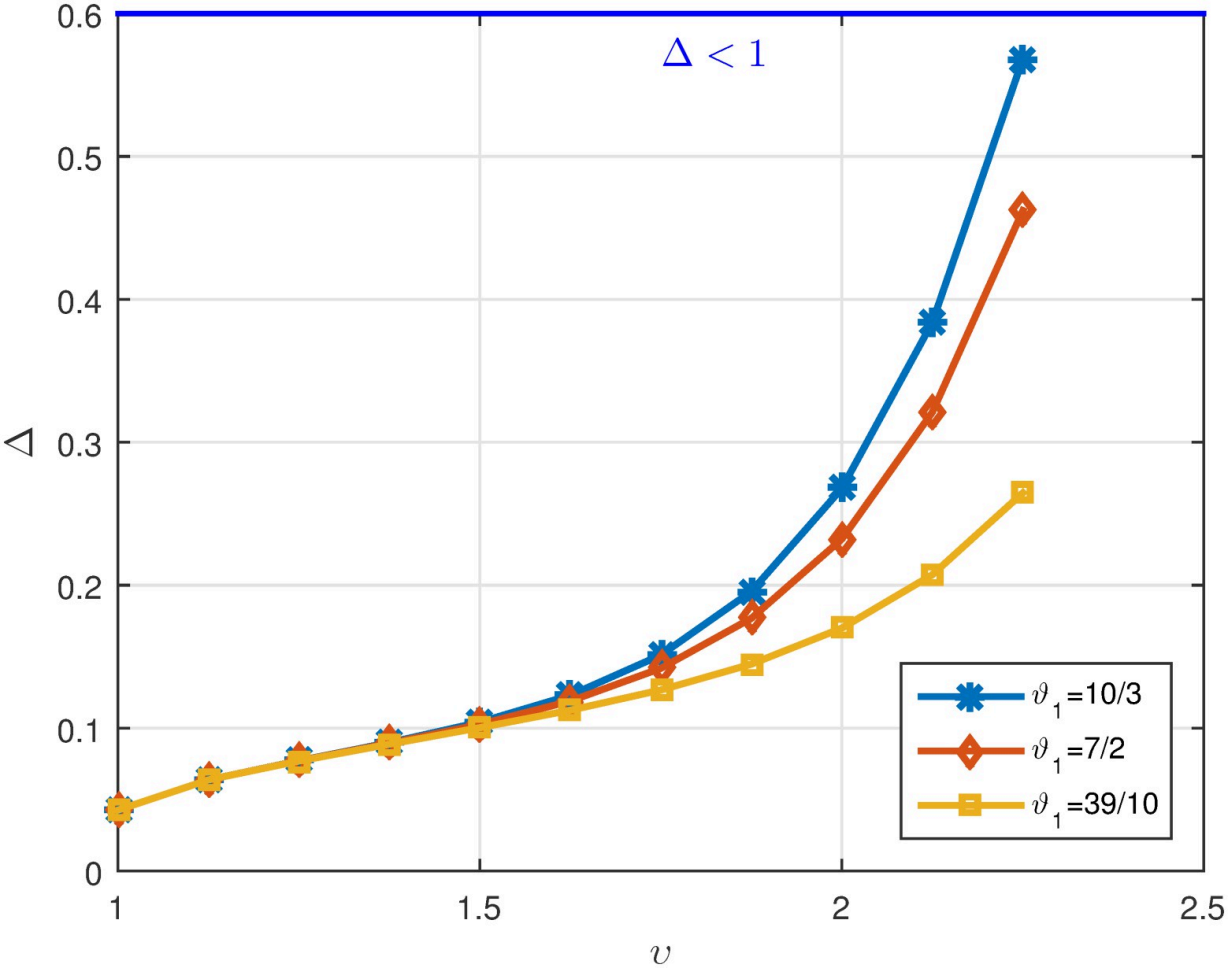
2D plots of Δ for three cases of *ϑ*_1_ when *υ* ∈ J in Example 5.1.

**Table 1:** Numerical values of ${\hat{\Lambda }}_{1}$, ${\hat{\Lambda }}_{2}$ and Δ for three cases of *ϑ*_1_ when *ρ* = *e*^*υ*/2^ in Example 5.1.

*υ*	$\vartheta_{1}=\frac{10}{3}$			$\vartheta_{1}=\frac{7}{2}$			$\vartheta_{1}=\frac{39}{10}$		
	${\hat{\Lambda }}_{1}$	${\hat{\Lambda }}_{2}$	Δ < 1	${\hat{\Lambda }}_{1}$	${\hat{\Lambda }}_{2}$	Δ < 1	${\hat{\Lambda }}_{1}$	${\hat{\Lambda }}_{2}$	Δ < 1
1.00	0.4163	0.0000	0.0426	0.4163	0.0000	0.0426	0.4163	0.0000	0.0426
1.13	0.6233	0.0000	0.0638	0.6233	0.0000	0.0638	0.6233	0.0000	0.0637
1.25	0.7519	0.0002	0.0771	0.7519	0.0001	0.0770	0.7519	0.0000	0.0769
1.38	0.8656	0.0011	0.0898	0.8656	0.0007	0.0894	0.8656	0.0002	0.0887
1.50	0.9724	0.0040	0.1041	0.9724	0.0027	0.1026	0.9724	0.0007	0.1003
1.63	1.0759	0.0112	0.1230	1.0759	0.0076	0.1189	1.0759	0.0021	0.1125
1.75	1.1777	0.0264	0.1511	1.1777	0.0184	0.1418	1.1777	0.0053	0.1266
1.88	1.2791	0.0559	0.1956	1.2791	0.0396	0.1768	1.2791	0.0120	0.1447
2.00	1.3808	0.1091	0.2677	1.3808	0.0785	0.2323	1.3808	0.0248	0.1699
2.13	1.4835	0.2001	0.3838	1.4835	0.1460	0.3210	1.4835	0.0479	0.2073
2.25	1.5877	0.3499	0.5682	1.5877	0.2584	0.4621	1.5877	0.0881	0.2646

In the example 5.2, we check the validity of Theorem 3.3 for four cases of function *ρ*(*υ*).

**Example 5.2**. *We consider the same* ρ-ABC FGLSLP (54) *defined in Example 5.1 as form*

DABCa18/5,ρ[1υDABCa2/3,ρ+135υcos(πυ)]ω(υ)=3tan-1υ(sinω(υ)+63)5(2+|υ|), (63)

*with* $\vartheta_{1}=\frac{18}{5}\in \left(3,4\right]$ *is fixed, but*

$$\begin{array}{c} \rho_{1}\left(\upsilon \right)=1.5^{\upsilon },\;\rho_{2}\left(\upsilon \right)=\upsilon ,\;\rho_{3}\left(\upsilon \right)=\text{ln}\;\upsilon ,\;\rho_{4}\left(\upsilon \right)=\sqrt{\upsilon }, \end{array}$$ (64)

*on domain* J = [1, 2.25], *under the same conditions* (56). *There we showed that assumptions* (G1)–(G3) *are valid. Just check the values of* ${\hat{\Lambda }}_{1}$, ${\hat{\Lambda }}_{2}$ *and* Δ *calculations again. In this case, by employing* Eq (25), *we obtain*

$$\begin{array}{cc} {\hat{\Lambda }}_{1} & =\frac{1-\vartheta_{2}}{\left|\Lambda \left(\vartheta_{2}\right)\right|}+\frac{\vartheta_{2}{\left({\hat{\rho }}_{a}\left(\upsilon \right)\right)}^{\vartheta_{2}}}{|\Lambda \left(\vartheta_{2}\right)|\;\Gamma \left(\vartheta_{2}+1\right)}\\ & =\frac{1}{3|\text{exp}\;\left({\left(\frac{2}{3}\right)}^{2}-\frac{2}{3}\right)|}+\frac{2{\left(\rho_{i}\left(b\right)-\rho_{i}\left(a\right)\right)}^{2/3}}{3|\text{exp}\;\left({\left(\frac{2}{3}\right)}^{2}-\frac{2}{3}\right)|\Gamma \left(\frac{5}{3}\right)}≃\{\begin{array}{cc} 1.3324, & \rho_{1}\left(\upsilon \right)=1.5^{\upsilon },\\ 1.4865, & \rho_{2}\left(\upsilon \right)=\upsilon ,\\ 1.2183, & \rho_{3}\left(\upsilon \right)=\text{ln}\;\upsilon ,\\ 0.9973, & \rho_{4}\left(\upsilon \right)=\sqrt{\upsilon }, \end{array} \end{array}$$ (65)

$$\begin{array}{c} {\hat{\Lambda }}_{2}≃\{\begin{array}{cc} 0.0449, & \rho_{1}\left(\upsilon \right)=1.5^{\upsilon },\\ 0.1180, & \rho_{2}\left(\upsilon \right)=\upsilon ,\\ 0.0197, & \rho_{3}\left(\upsilon \right)=\text{ln}\;\upsilon ,\\ 0.0027, & \rho_{4}\left(\upsilon \right)=\sqrt{\upsilon }, \end{array} \end{array}$$ (66)

*and by using* Eq (24) *we get*

$$\begin{array}{c} \Delta =\frac{\gamma^{*}\;{\hat{\Lambda }}_{1}+\left\|\alpha \right\|\;{\hat{\Lambda }}_{2}}{\mu_{*}}≃\{\begin{array}{cc} 0.1883, & \rho_{1}\left(\upsilon \right)=1.5^{\upsilon },\\ 0.2889, & \rho_{2}\left(\upsilon \right)=\upsilon ,\\ 0.1474, & \rho_{3}\left(\upsilon \right)=\text{ln}\;\upsilon ,\\ 0.1051, & \rho_{4}\left(\upsilon \right)=\sqrt{\upsilon }, \end{array}\}<1. \end{array}$$ (67)

**Fig 3:**
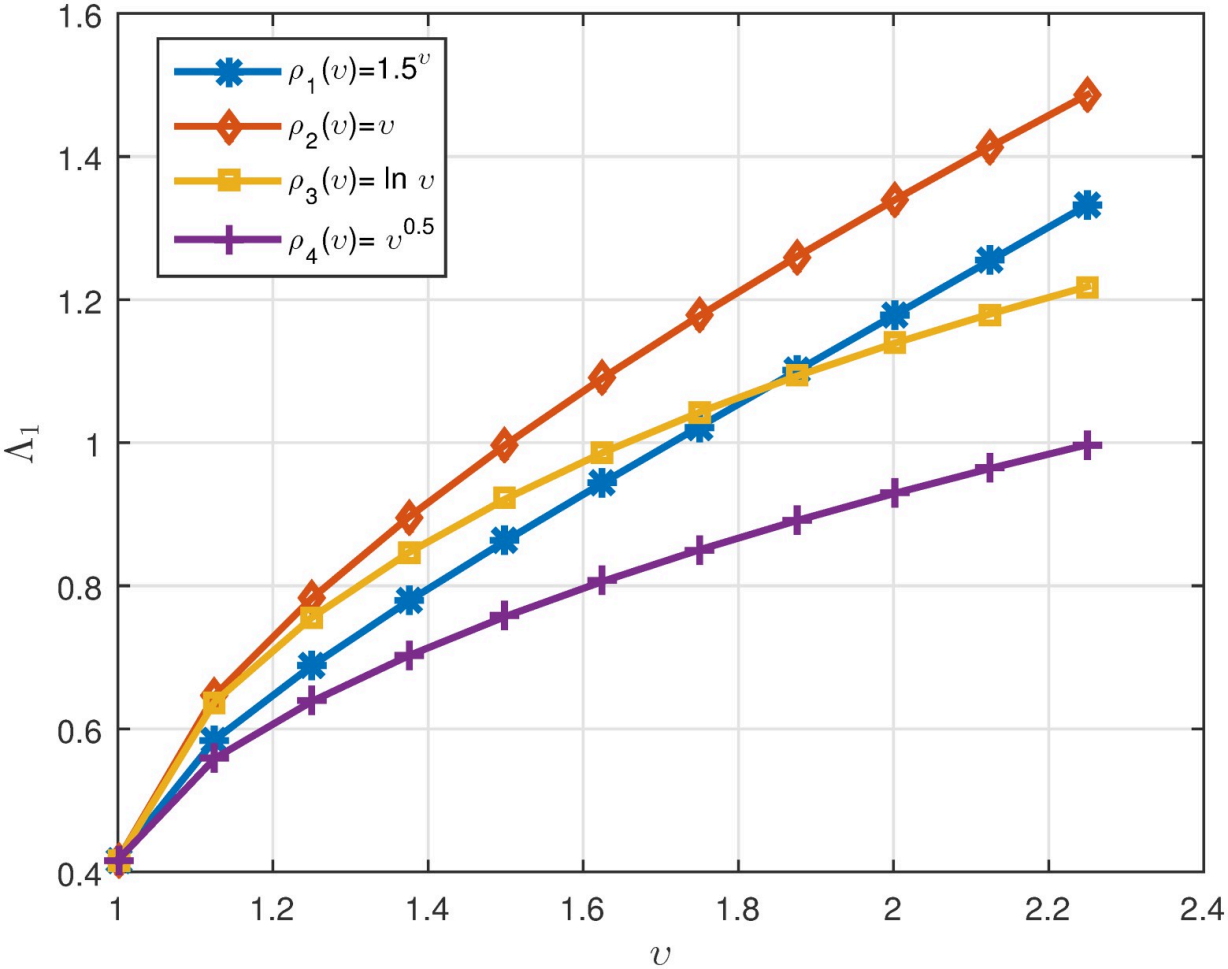
2D plots of ${\hat{\Lambda }}_{1}$ for four cases of *ρ*(*υ*) when *υ* ∈ J in Example 5.2.

**Fig 4:**
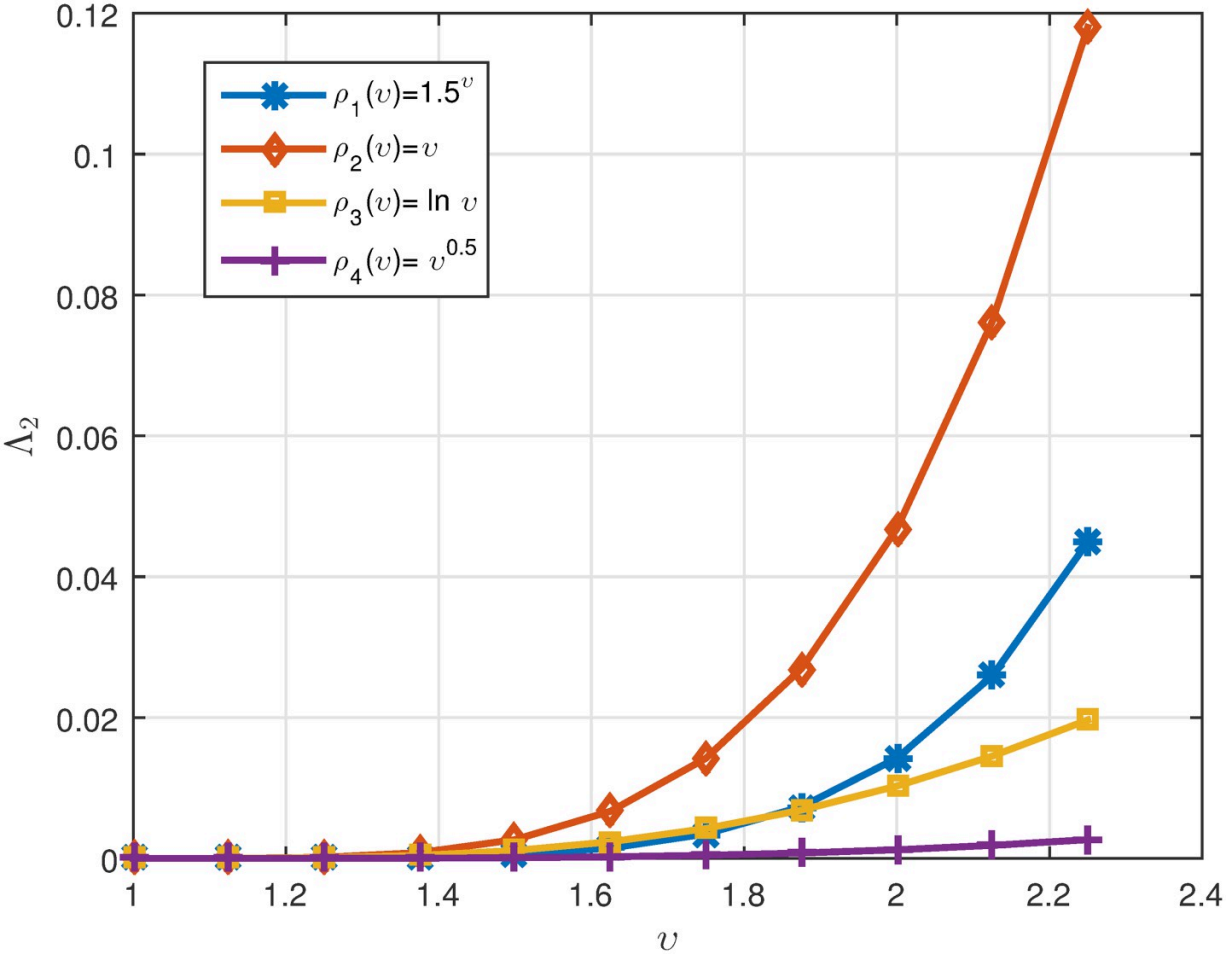
2D plots of ${\hat{\Lambda }}_{2}$ for four cases of *ρ*(*υ*) when *υ* ∈ J in Example 5.2.

**Fig 5:**
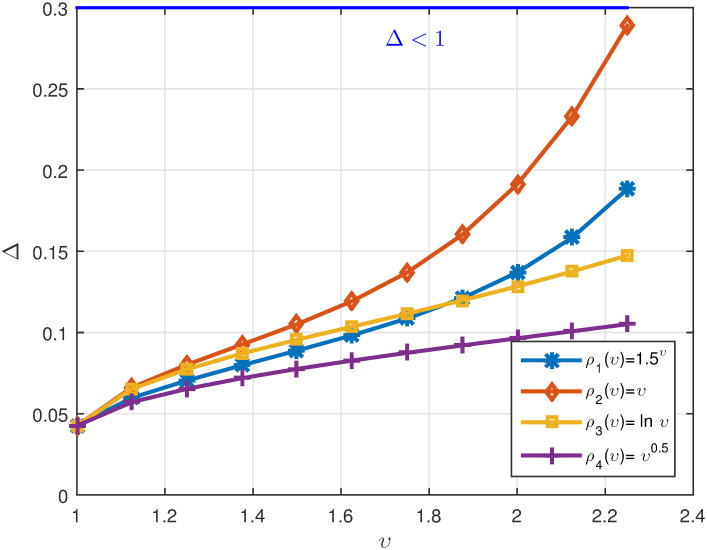
2D plots of Δ for four cases of *ρ*(*υ*) when *υ* ∈ J in Example 5.2.

*We plot the numerical results in* Figs 3–5 *for four case of function* *ρ*(*υ*), *respectively. Also, one can see the data in* Tables 2 *and* 3 *for different cases of the function* *ρ*(*υ*) *on* *υ* ∈ [1, 2.25]. *Hence, conditions of Theorem 3.3 are hold, and so, the* ρ-ABC FGLSLP (63) *admits unique solution on domain* J = [1, 2.25] *for all of four cases of ρ*(*υ*). *To find the appropriate value for* *r, we use* $g_{0}=\frac{\sqrt[{3}]{6}}{\sqrt{5}}\;{\text{tan}}^{-1}\;\left(2.25\right)$ *and relation* (28):

$$\begin{array}{cc} {\hat{\Lambda }}_{3} & =\sum_{k=0}^{m}\frac{|\xi_{k}|}{k!}\left(\frac{\left(1-\vartheta_{2}\right){\left({\hat{\rho }}_{a}\left(\upsilon \right)\right)}^{k}}{\left|\Lambda \left(\vartheta_{2}\right)\right|}+\frac{\vartheta_{2}\Gamma \left(k+1\right){\left({\hat{\rho }}_{a}\left(\upsilon \right)\right)}^{k+\vartheta_{2}}}{|\Lambda \left(\vartheta_{2}\right)|\;\Gamma \left(\vartheta_{2}+k+1\right)}\right)\\ & =\frac{4}{19}[\frac{1}{3|\text{exp}\;\left({\left(\frac{2}{3}\right)}^{2}-\frac{2}{3}\right)|}+\frac{2{\left({\hat{\rho }}_{a}\left(b\right)\right)}^{2/3}}{3|\text{exp}\;\left({\left(\frac{-7}{3}\right)}^{2}+\frac{7}{3}\right)|\Gamma \left(\frac{5}{3}\right)}]\\ & \;+\frac{7}{13}[\frac{{\hat{\rho }}_{a}\left(b\right)}{3|\text{exp}\;\left({\left(\frac{2}{3}\right)}^{2}-\frac{2}{3}\right)|}+\frac{2\Gamma \left(2\right){\left({\hat{\rho }}_{a}\left(b\right)\right)}^{5/3}}{3|\text{exp}\;\left({\left(\frac{2}{3}\right)}^{2}-\frac{2}{3}\right)|\Gamma \left(\frac{8}{3}\right)}]\\ & \;+\frac{8}{33}[\frac{{\left({\hat{\rho }}_{a}\left(b\right)\right)}^{2}}{3|\text{exp}\;\left({\left(\frac{2}{3}\right)}^{2}-\frac{2}{3}\right)|}+\frac{2\Gamma \left(3\right){\left({\hat{\rho }}_{a}\left(b\right)\right)}^{8/3}}{3|\text{exp}\;\left({\left(\frac{2}{3}\right)}^{2}-\frac{2}{3}\right)|\Gamma \left(\frac{11}{3}\right)}]\\ & \;+\frac{14}{27}[\frac{{\left({\hat{\rho }}_{a}\left(b\right)\right)}^{3}}{3|\text{exp}\;\left({\left(\frac{2}{3}\right)}^{2}-\frac{2}{3}\right)|}+\frac{2\Gamma \left(4\right){\left({\hat{\rho }}_{a}\left(b\right)\right)}^{11/3}}{3|\text{exp}\;\left({\left(\frac{2}{3}\right)}^{2}-\frac{2}{3}\right)|\Gamma \left(\frac{14}{3}\right)}]\\ & ≃\{\begin{array}{cc} 1.3715, & \rho_{1}\left(\upsilon \right)=1.5^{\upsilon },\\ 2.1860, & \rho_{2}\left(\upsilon \right)=\upsilon ,\\ 0.9690, & \rho_{3}\left(\upsilon \right)=\text{ln}\;\upsilon ,\\ 0.4978, & \rho_{4}\left(\upsilon \right)=\sqrt{\upsilon }, \end{array} \end{array}$$

$$\begin{array}{cc} {\hat{\Lambda }}_{4} & =|\eta_{1}|+\frac{|1-\vartheta_{2}|}{\left|\Lambda (\vartheta_{2})\right|}[\frac{|\eta_{1}\left|\;|\gamma \left(a\right)\right|}{\left|\mu (a)\right|}+\frac{|\xi_{0}|}{\left|\mu (a)\right|}]\\ & =|\frac{-14}{\sqrt{129}}|+\frac{\frac{1}{3}}{\left|\text{exp}\;\left({\left(\frac{2}{3}\right)}^{2}-\frac{2}{3}\right)\right|}[|\frac{-14}{\sqrt{129}}|(\frac{-1}{35})+|\frac{-4}{19}|]≃1.3349. \end{array}$$ (68)

**Table 2:** Numerical values of ${\hat{\Lambda }}_{1}$, ${\hat{\Lambda }}_{2}$ and Δ for four cases of *ρ* in Example 5.2.

*υ*	*ρ*_1_(*υ*) = 1.5^*υ*^			*ρ*_2_(*υ*) = *υ*		
	${\hat{\Lambda }}_{1}$	${\hat{\Lambda }}_{2}$	Δ < 1	${\hat{\Lambda }}_{1}$	${\hat{\Lambda }}_{2}$	Δ < 1
1.00	0.4163	0.0000	0.0426	0.4163	0.0000	0.0426
1.13	0.5846	0.0000	0.0598	0.6468	0.0000	0.0662
1.25	0.6881	0.0000	0.0704	0.7823	0.0002	0.0802
1.38	0.7787	0.0001	0.0798	0.8959	0.0008	0.0926
1.50	0.8630	0.0005	0.0889	0.9973	0.0027	0.1051
1.63	0.9438	0.0015	0.0982	1.0905	0.0067	0.1193
1.75	1.0226	0.0035	0.1086	1.1776	0.0142	0.1369
1.88	1.1004	0.0073	0.1210	1.2600	0.0269	0.1601
2.00	1.1776	0.0142	0.1369	1.3385	0.0468	0.1912
2.13	1.2548	0.0259	0.1584	1.4139	0.0762	0.2330
2.25	1.3324	0.0449	0.1883	1.4865	0.1180	0.2889

**Table 3:** Numerical values of ${\hat{\Lambda }}_{1}$, ${\hat{\Lambda }}_{2}$ and Δ for four cases of *ρ* in Example 5.2.

*υ*	*ρ*_3_(*υ*) = ln *υ*			$\rho_{4}\left(\upsilon \right)=\sqrt{\upsilon }$		
	${\hat{\Lambda }}_{1}$	${\hat{\Lambda }}_{2}$	Δ < 1	${\hat{\Lambda }}_{1}$	${\hat{\Lambda }}_{2}$	Δ < 1
1.00	0.4163	0.0000	0.0426	0.4163	0.0000	0.0426
1.13	0.6379	0.0000	0.0653	0.5587	0.0000	0.0571
1.25	0.7556	0.0001	0.0774	0.6382	0.0000	0.0653
1.38	0.8464	0.0004	0.0870	0.7022	0.0000	0.0719
1.50	0.9215	0.0011	0.0956	0.7572	0.0001	0.0776
1.63	0.9860	0.0024	0.1036	0.8061	0.0002	0.0827
1.75	1.0426	0.0042	0.1115	0.8503	0.0004	0.0875
1.88	1.0931	0.0069	0.1197	0.8910	0.0008	0.0920
2.00	1.1386	0.0103	0.1284	0.9288	0.0012	0.0964
2.13	1.1801	0.0145	0.1375	0.9641	0.0018	0.1007
2.25	1.2183	0.0197	0.1474	0.9973	0.0027	0.1051

Table 4 *shows the obtained numerical results. Because the parameter* ${\hat{\Lambda }}_{4}$ *is not dependent on the function* *ρ*(*υ*), *it is displayed in one column in* Table 4. *Thus*

$$\begin{array}{cc} r & \geq [\frac{g_{0}}{\mu_{*}}{\hat{\Lambda }}_{2}+\frac{1}{\mu_{*}}{\hat{\Lambda }}_{3}+{\hat{\Lambda }}_{4}]\;{\left[1-(\frac{\gamma^{*}}{\mu_{*}}{\hat{\Lambda }}_{1}+\frac{\left\|\alpha \right\|}{\mu_{*}}{\hat{\Lambda }}_{2})\right]}^{-1}\\ & ≃\{\begin{array}{cc} 5.5633, & \rho_{1}\left(\upsilon \right)=1.5^{\upsilon },\\ 9.1436, & \rho_{2}\left(\upsilon \right)=\upsilon ,\\ 4.1715, & \rho_{3}\left(\upsilon \right)=\text{ln}\;\upsilon ,\\ 2.7495, & \rho_{4}\left(\upsilon \right)=\sqrt{\upsilon }. \end{array} \end{array}$$ (69)

**Fig 6:**
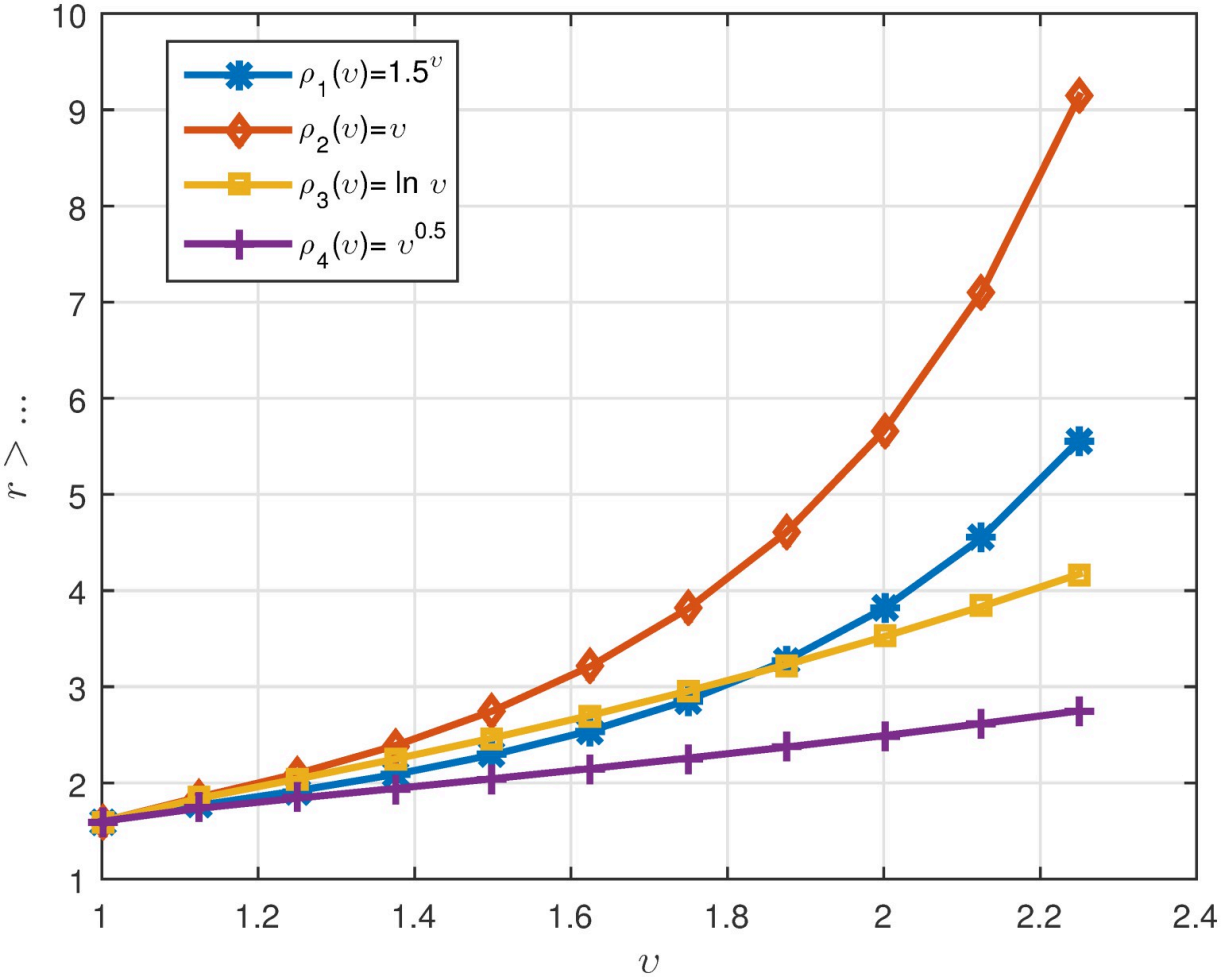
Representation of suitable value for *r* for different cases of the function *ρ* in Example 5.2.

*The curve of the minimum suitable value for r is drawn in* Fig 6.

**Table 4:** Numerical values of ${\hat{\Lambda }}_{3}$, ${\hat{\Lambda }}_{4}$ and *r* for four cases of *ρ* in Example 5.2.

*υ*	${\hat{\Lambda }}_{3}$ , (*ρ*_*i*_(*υ*) = …)				${\hat{\Lambda }}_{4}$	*r* > …, (*ρ*_*i*_(*υ*) = …)			
	1.5^*υ*^	*υ*	ln *υ*	$\sqrt{\upsilon }$		1.5^*υ*^	*υ*	ln *υ*	$\sqrt{\upsilon }$
1.00	0.0876	0.0876	0.0876	0.0876	1.3349	1.6003	1.6003	1.6003	1.6003
1.13	0.1456	0.1760	0.1713	0.1345	1.3349	1.7684	1.8536	1.8404	1.7368
1.25	0.1992	0.2636	0.2435	0.1714	1.3349	1.9183	2.0964	2.0410	1.8409
1.38	0.2608	0.3685	0.3184	0.2078	1.3349	2.0887	2.3868	2.2479	1.9421
1.50	0.3345	0.4978	0.3975	0.2447	1.3349	2.2923	2.7495	2.4675	2.0443
1.63	0.4247	0.6581	0.4814	0.2827	1.3349	2.5435	3.2130	2.7029	2.1493
1.75	0.5369	0.8564	0.5699	0.3222	1.3349	2.8611	3.8140	2.9560	2.2583
1.88	0.6780	1.1001	0.6632	0.3633	1.3349	3.2718	4.6041	3.2282	2.3722
2.00	0.8564	1.3974	0.7610	0.4062	1.3349	3.8140	5.6596	3.5207	2.4916
2.13	1.0830	1.7564	0.8630	0.4510	1.3349	4.5464	7.1026	3.8348	2.6172
2.25	1.3715	2.1860	0.9690	0.4978	1.3349	5.5633	9.1436	4.1715	2.7495

*In this example, all parts of Theorem 3.3 were examined along with its proof. The curves drawn in* Fig 6 *show that for the linear function* *ρ*(*υ*) = *υ*, *we should choose a value of r greater than* 9.2.

In the next example, we consider the extracted results for changes in order *ϑ*_2_, and we show that in addition to Theorem 3.3, Theorem 3.4 also confirms the existence of the solution.

**Example 5.3**. *We consider the following* ρ-ABC FGLSLP (54) *with a few changes which is defined in Example 5.1 as form*

DABCa19/5,υ/3[2υDABCaϑ2,υ/3+υcos(πυ)]ω(υ)=3tan-1υ(sinω(υ)+63)5(2+|υ|), (70)

*with order* $\vartheta_{1}=\frac{19}{5}\in \left(3,4\right]$ *and* $\rho \left(\upsilon \right)=\frac{\upsilon }{3}$ *are fixed but*

$$\begin{array}{c} \vartheta_{2}=\{\frac{8}{11},\;\frac{9}{11},\;\frac{10}{11},\;1\}\subset \left(0,1\right], \end{array}$$ (71)

*on domain* J = [1, 2.25], *under conditions* $\omega \left(1\right)=-\frac{14}{\sqrt{129}}$ *and*

[2DABCaϑ2,υ/3-1]υ/3(0)ω(υ)|υ=1=-418,[2DABCaϑ2,υ/3-1]υ/3(1)ω(υ)|υ=1=417,[2DABCaϑ2,υ/3-1]υ/3(2)ω(υ)|υ=1=-1033,[2DABCaϑ2,υ/3-1]υ/3(3)ω(υ)|υ=1=289.

*There we showed that assumptions* (G1)–(G3) *are valid. We just check the values of* ${\hat{\Lambda }}_{1}$, ${\hat{\Lambda }}_{2}$ *and* Δ *calculations again. Then, we calculate the values of* ${\hat{\Lambda }}_{3}$, ${\hat{\Lambda }}_{4}$, *Inequality* (30) *and* P. *In this case*, Eq (25) *implies that*

$$\begin{array}{c} {\hat{\Lambda }}_{1}=\frac{1-\vartheta_{2}}{\left|\Lambda (\vartheta_{2})\right|}+\frac{\vartheta_{2}{\left({\hat{\rho }}_{a}\left(\upsilon \right)\right)}^{\vartheta_{2}}}{|\Lambda \left(\vartheta_{2}\right)|\;\Gamma \left(\vartheta_{2}+1\right)}≃\{\begin{array}{cc} 0.3258, & \vartheta_{2}=\frac{8}{11},\\ 0.3545, & \vartheta_{2}=\frac{9}{11},\\ 0.3893, & \vartheta_{2}=\frac{10}{11},\\ 0.4167, & \vartheta_{2}=1, \end{array} \end{array}$$ (72)

$$\begin{array}{cc} {\hat{\Lambda }}_{2} & ≃\{\begin{array}{cc} 0.0030, & \vartheta_{2}=\frac{8}{11},\\ 0.0023, & \vartheta_{2}=\frac{9}{11},\\ 0.0016, & \vartheta_{2}=\frac{10}{11},\\ 0.0010, & \vartheta_{2}=1, \end{array} \end{array}$$ (73)

*and by using* Eq (24) *we get*

$$\begin{array}{c} \Delta =\frac{\gamma^{*}\;{\hat{\Lambda }}_{1}+\left\|\alpha \right\|\;{\hat{\Lambda }}_{2}}{\mu_{*}}≃\{\begin{array}{cc} 0.5849, & \vartheta_{2}=\frac{8}{11},\\ 0.6359, & \vartheta_{2}=\frac{9}{11},\\ 0.6977, & \vartheta_{2}=\frac{10}{11},\\ 0.7464, & \vartheta_{2}=1, \end{array}\}<1. \end{array}$$ (74)

**Fig 7:**
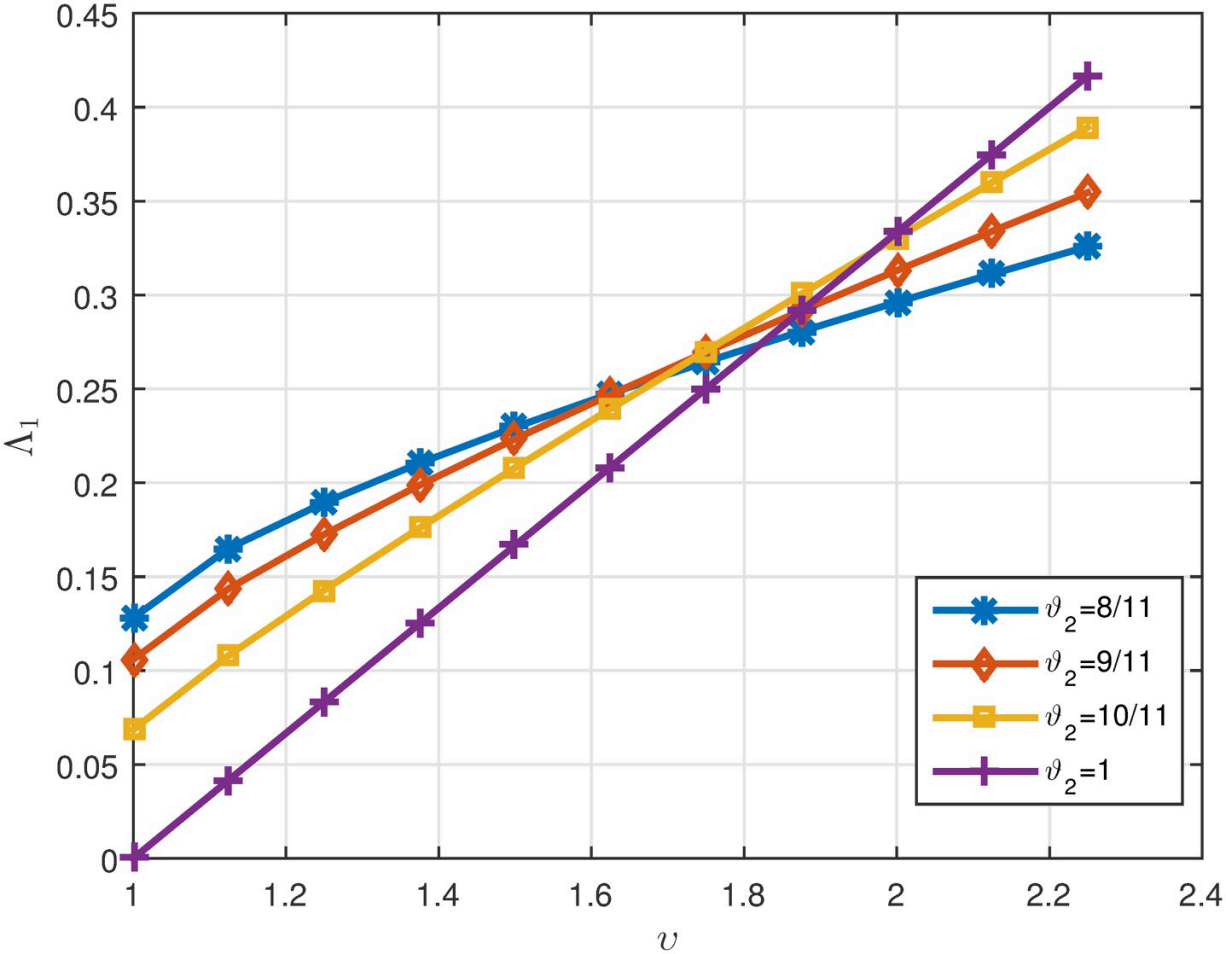
2D plots of ${\hat{\Lambda }}_{1}$ for four cases of *ϑ*_2_ in Example 5.3 for *υ* ∈ J.

**Fig 8:**
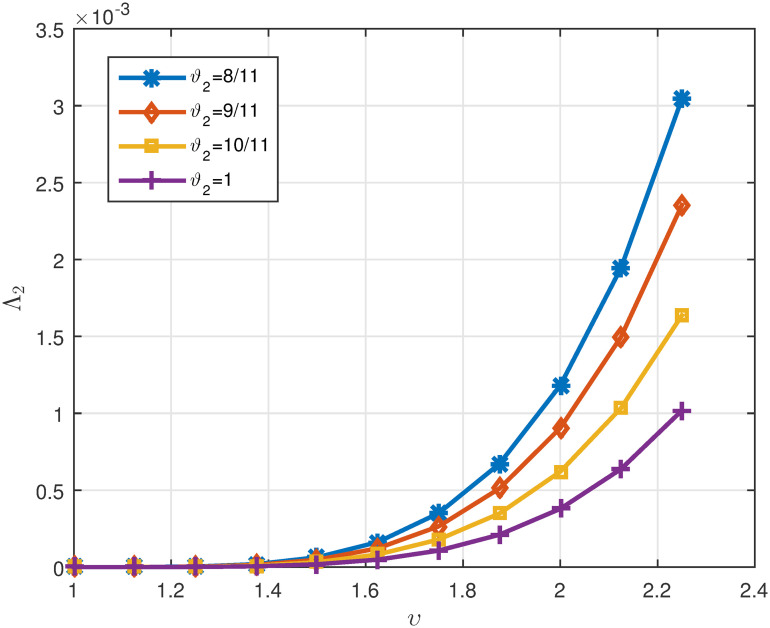
2D plots of ${\hat{\Lambda }}_{2}$ for four cases of *ϑ*_2_ in Example 5.3 for *υ* ∈ J.

**Fig 9:**
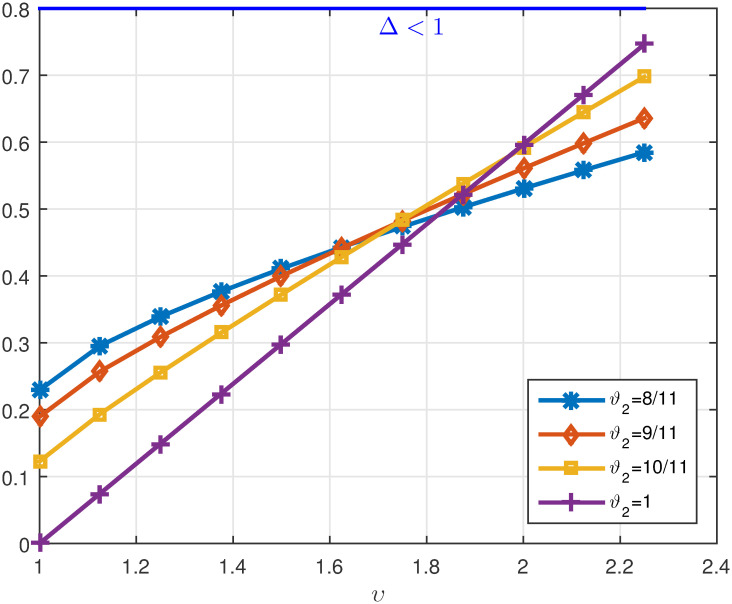
2D plots of Δ for four cases of *ϑ*_2_ in Example 5.3 for *υ* ∈ J.

Tables 5 *and* 6 *show the numerical results of* ${\hat{\Lambda }}_{1}$, ${\hat{\Lambda }}_{2}$ *and* Δ *for* *υ* ∈ [1, 2.25] *and for different cases of* *ϑ*_2_. *These values are also shown in* Figs 7–9 *for four cases of* *ϑ*_2_, *respectively. So, conditions of Theorem 3.3 are verified, which this ensure that for all of four cases of* *ϑ*_2_, *the* ρ-ABC FGLSLP (70) *admits unique solution on domain* J = [1, 2.25]. *Now, to find the suitable value for* P, *we use* $\left\|\alpha \right\|=\frac{3}{\sqrt{5}\left(2+|\upsilon |\right)}\;{\text{tan}}^{-1}\;\upsilon ≃0.5154$ *and relation* (28):

$$\begin{array}{c} {\hat{\Lambda }}_{3}=\sum_{k=0}^{m}\frac{|\xi_{k}|}{k!}(\frac{\left(1-\vartheta_{2}\right){\left({\hat{\rho }}_{a}\left(\upsilon \right)\right)}^{k}}{\left|\Lambda (\vartheta_{2})\right|}+\frac{\vartheta_{2}\Gamma \left(k+1\right){\left({\hat{\rho }}_{a}\left(\upsilon \right)\right)}^{k+\vartheta_{2}}}{|\Lambda \left(\vartheta_{2}\right)|\;\Gamma \left(\vartheta_{2}+k+1\right)})≃\{\begin{array}{cc} 0.6772, & \vartheta_{2}=\frac{8}{11},\\ 0.6831, & \vartheta_{2}=\frac{9}{11},\\ 0.6746, & \vartheta_{2}=\frac{10}{11},\\ 0.6086, & \vartheta_{2}=1, \end{array} \end{array}$$ (75)

$$\begin{array}{c} {\hat{\Lambda }}_{4}=|\eta_{1}|+\frac{|1-\vartheta_{2}|}{\left|\Lambda (\vartheta_{2})\right|}[\frac{|\eta_{1}\left|\;|\gamma \left(a\right)\right|}{\left|\mu (a)\right|}+\frac{|\xi_{0}|}{\left|\mu (a)\right|}]≃\{\begin{array}{cc} 1.3258, & \vartheta_{2}=\frac{8}{11},\\ 1.3097, & \vartheta_{2}=\frac{9}{11},\\ 1.2825, & \vartheta_{2}=\frac{10}{11},\\ 1.2326, & \vartheta_{2}=1. \end{array} \end{array}$$ (76)

**Table 5:** Numerical values of ${\hat{\Lambda }}_{1}$, ${\hat{\Lambda }}_{2}$ and Δ for four cases of *ϑ*_2_ in Example 5.3.

*υ*	$\vartheta_{2}=\frac{8}{11}$			$\vartheta_{2}=\frac{9}{11}$		
	${\hat{\Lambda }}_{1}$	${\hat{\Lambda }}_{2}$	Δ < 1	${\hat{\Lambda }}_{1}$	${\hat{\Lambda }}_{2}$	Δ < 1
1.00	0.1281	0.00000	0.2293	0.1059	0.00000	0.1895
1.13	0.1651	0.00000	0.2956	0.1437	0.00000	0.2572
1.25	0.1894	0.00000	0.3390	0.1725	0.00000	0.3088
1.38	0.2104	0.00002	0.3767	0.1987	0.00001	0.3557
1.50	0.2296	0.00006	0.4110	0.2234	0.00005	0.3998
1.63	0.2475	0.00016	0.4431	0.2469	0.00012	0.4420
1.75	0.2644	0.00035	0.4735	0.2696	0.00027	0.4827
1.88	0.2806	0.00067	0.5026	0.2916	0.00051	0.5222
2.00	0.2962	0.00118	0.5308	0.3130	0.00090	0.5608
2.13	0.3112	0.00195	0.5581	0.3340	0.00150	0.5986
2.25	0.3258	0.00304	0.5849	0.3545	0.00235	0.6359

**Table 6:** Numerical values of ${\hat{\Lambda }}_{1}$, ${\hat{\Lambda }}_{2}$ and Δ for four cases of *ϑ*_2_ in Example 5.3.

*υ*	$\vartheta_{2}=\frac{10}{11}$			*ϑ*_2_ = 1		
	${\hat{\Lambda }}_{1}$	${\hat{\Lambda }}_{2}$	Δ < 1	${\hat{\Lambda }}_{1}$	${\hat{\Lambda }}_{2}$	Δ < 1
1.00	0.0686	0.00000	0.1228	0.0000	0.00000	0.0000
1.13	0.1081	0.00000	0.1935	0.0417	0.00000	0.0746
1.25	0.1428	0.00000	0.2557	0.0833	0.00000	0.1492
1.38	0.1759	0.00001	0.3149	0.1250	0.00001	0.2237
1.50	0.2080	0.00003	0.3723	0.1667	0.00002	0.2983
1.63	0.2394	0.00008	0.4285	0.2083	0.00005	0.3729
1.75	0.2702	0.00018	0.4837	0.2500	0.00011	0.4475
1.88	0.3005	0.00035	0.5380	0.2917	0.00021	0.5222
2.00	0.3304	0.00062	0.5918	0.3333	0.00038	0.5968
2.13	0.3600	0.00104	0.6450	0.3750	0.00064	0.6716
2.25	0.3893	0.00164	0.6977	0.4167	0.00102	0.7464

**Fig 10:**
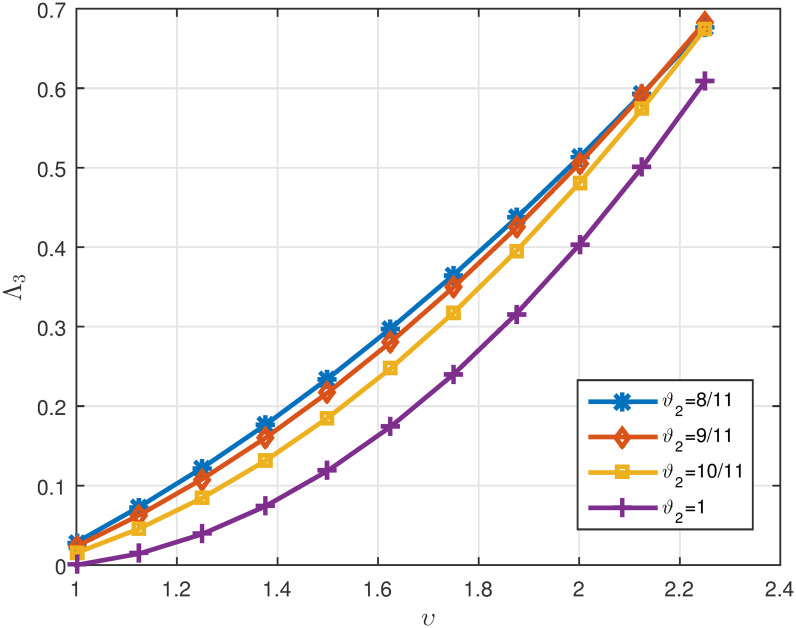
2D plots of ${\hat{\Lambda }}_{3}$ for four cases of *ϑ*_2_ in Example 5.3 for *υ* ∈ J.

**Fig 11:**
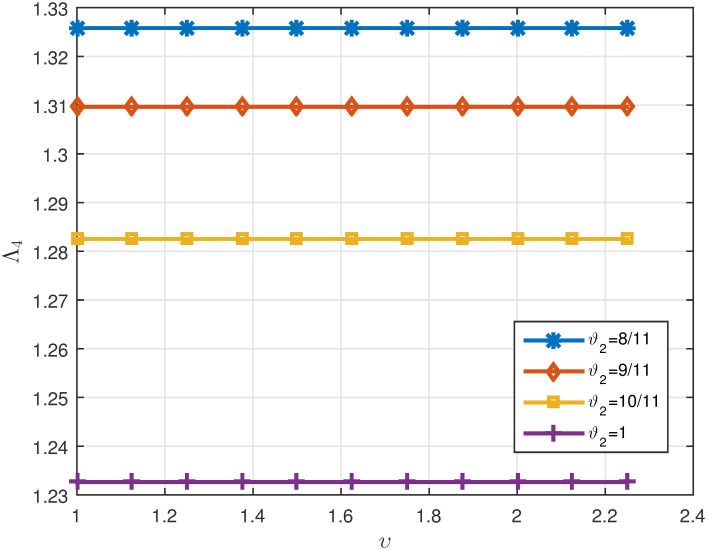
2D plots of ${\hat{\Lambda }}_{4}$ for four cases of *ϑ*_2_ in Example 5.3 for *υ* ∈ J.

**Fig 12:**
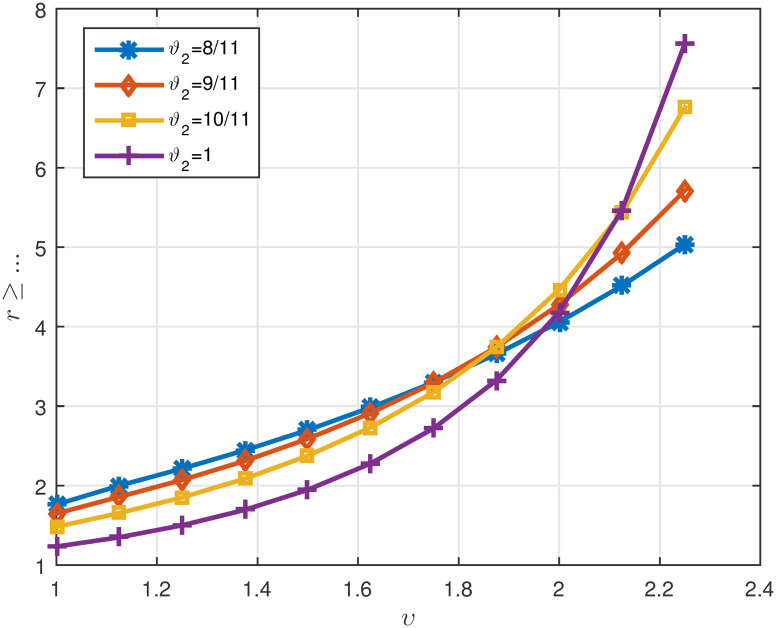
2D plots of suitable values *r* for four cases of *ϑ*_2_ in Example 5.3 for *υ* ∈ J.

Tables 7 *and* 8, *show the obtained numerical results for* ${\hat{\Lambda }}_{3}$, ${\hat{\Lambda }}_{4}$ *and* $\frac{1}{\mu_{*}}\gamma^{*}{\hat{\Lambda }}_{1}$. *It can be seen in* Figs 10–12 *that by reducing the order of the derivative* *ϑ*_2_ *to less than one, the value of* $\frac{1}{\mu_{*}}\gamma^{*}{\hat{\Lambda }}_{1}$ *should also increase, but it is less than 1. Indeed, Inequality* (30) *holds*. *Thus*,

$$\begin{array}{c} P\geq \left[\frac{1}{\mu_{*}}{\hat{\Lambda }}_{3}+{\hat{\Lambda }}_{4}\right]{\left[1-(\frac{\gamma^{*}}{\mu_{*}}{\hat{\Lambda }}_{1}+\frac{\left\|\alpha \right\|}{\mu_{*}}{\hat{\Lambda }}_{2})\right]}^{-1}≃\{\begin{array}{cc} 5.0288, & \vartheta_{2}=\frac{8}{11},\\ 5.7073, & \vartheta_{2}=\frac{9}{11},\\ 6.7542, & \vartheta_{2}=\frac{10}{11},\\ 7.5593, & \vartheta_{2}=1. \end{array} \end{array}$$ (77)

**Table 7:** Numerical values of ${\hat{\Lambda }}_{3}$, ${\hat{\Lambda }}_{4}$ and $\frac{1}{\mu_{*}}\gamma^{*}{\hat{\Lambda }}_{1}<1$ for four cases of *ϑ*_2_ in Example 5.3.

*υ*	$\vartheta_{2}=\frac{8}{11}$				$\vartheta_{2}=\frac{9}{11}$			
	${\hat{\Lambda }}_{3}$	${\hat{\Lambda }}_{4}$	$\frac{\gamma^{*}{\hat{\Lambda }}_{1}}{\mu_{*}}<1$	P ≥ …	${\hat{\Lambda }}_{3}$	${\hat{\Lambda }}_{4}$	$\frac{\gamma^{*}{\hat{\Lambda }}_{1}}{\mu_{*}}<1$	P ≥ …
1.00	0.0285	1.3258	0.2293	1.7617	0.0235	1.3097	0.1895	1.6486
1.13	0.0732	1.3258	0.2956	1.9990	0.0629	1.3097	0.2572	1.8583
1.25	0.1221	1.3258	0.3390	2.2137	0.1081	1.3097	0.3088	2.0706
1.38	0.1760	1.3258	0.3767	2.4447	0.1595	1.3097	0.3557	2.3113
1.50	0.2346	1.3258	0.4110	2.6990	0.2171	1.3097	0.3998	2.5889
1.63	0.2976	1.3258	0.4430	2.9818	0.2805	1.3097	0.4419	2.9124
1.75	0.3651	1.3258	0.4733	3.2982	0.3497	1.3097	0.4825	3.2919
1.88	0.4368	1.3258	0.5022	3.6537	0.4246	1.3097	0.5219	3.7406
2.00	0.5128	1.3258	0.5301	4.0549	0.5051	1.3097	0.5603	4.2756
2.13	0.5929	1.3258	0.5570	4.5099	0.5913	1.3097	0.5978	4.9204
2.25	0.6772	1.3258	0.5831	5.0288	0.6831	1.3097	0.6345	5.7073

**Table 8:** Numerical values of ${\hat{\Lambda }}_{3}$, ${\hat{\Lambda }}_{4}$ and $\frac{1}{\mu_{*}}\gamma^{*}{\hat{\Lambda }}_{1}<1$ for four cases of *ϑ*_2_ in Example 5.3.

*υ*	$\vartheta_{2}=\frac{10}{11}$				*ϑ*_2_ = 1			
	${\hat{\Lambda }}_{3}$	${\hat{\Lambda }}_{4}$	$\frac{\gamma^{*}{\hat{\Lambda }}_{1}}{\mu_{*}}<1$	P ≥ …	${\hat{\Lambda }}_{3}$	${\hat{\Lambda }}_{4}$	$\frac{\gamma^{*}{\hat{\Lambda }}_{1}}{\mu_{*}}<1$	P ≥ …
1.00	0.0152	1.2825	0.1228	1.4816	0.0000	1.2326	0.0000	1.2326
1.13	0.0458	1.2825	0.1935	1.6543	0.0143	1.2326	0.0746	1.3494
1.25	0.0843	1.2825	0.2557	1.8505	0.0389	1.2326	0.1492	1.5001
1.38	0.1308	1.2825	0.3149	2.0869	0.0737	1.2326	0.2237	1.6947
1.50	0.1852	1.2825	0.3723	2.3754	0.1187	1.2326	0.2983	1.9470
1.63	0.2475	1.2825	0.4285	2.7312	0.1741	1.2326	0.3729	2.2780
1.75	0.3174	1.2825	0.4836	3.1755	0.2399	1.2326	0.4475	2.7196
1.88	0.3951	1.2825	0.5378	3.7385	0.3161	1.2326	0.5220	3.3239
2.00	0.4806	1.2825	0.5914	4.4659	0.4029	1.2326	0.5966	4.1818
2.13	0.5737	1.2825	0.6444	5.4303	0.5004	1.2326	0.6712	5.4671
2.25	0.6746	1.2825	0.6968	6.7542	0.6086	1.2326	0.7458	7.5593

**Fig 13:**
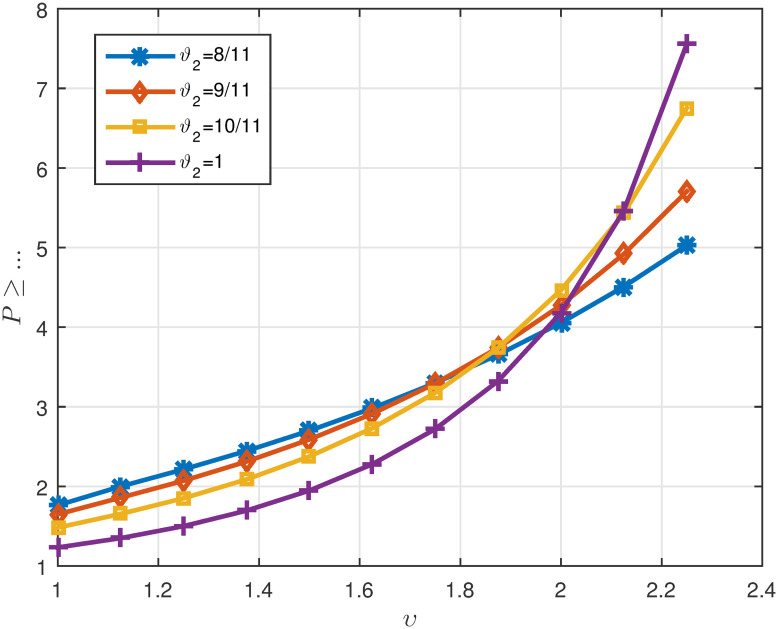
Representation of suitable value for P ≥ … for different cases of *ϑ*_2_ in Example 5.3.

*The curve of the minimum suitable value for* P *is drawn in* Fig 13. *In this example, all parts of Theorem 3.4 were examined along with its proof. The curves drawn in* Fig 13 *show that when* *ϑ*_2_ *is equal to* 1, P *can have its lowest value*.

## 6 Conclusion

In this paper, we investigated a new class of ρ-ABC higher order FGLSLP with the ρ-ABC fractional derivatives. To confirm the sufficient conditions of the existence and uniqueness criterion, we used the valid Krasnoselskii and Banach FPT. Furthermore, we discussed the UH, the generalized UH, the UHR, and the generalized UHR stabilities for the proposed problem (3). In the end, we presented four comprehensive examples, supported by graphics and tables for different cases of fractional orders *ϑ*_1_, *ϑ*_2_, and respected function *ρ*, to checking our major findings. Of course, our results are valid to the FGLSLP in the sense of many types of derivatives. For instance ABC- fractional derivatives for *ρ*(*υ*) = *υ*, Hadamard version for *ρ*(*υ*) = ln(*υ*), Katugampola form for *ρ*(*υ*) = *υ*^*p*^, *p* > 0, and ordinary derivatives for *ρ*(*υ*) = *υ* and $\vartheta_{1}=m\in N$, *ϑ*_2_ = 1.
